# Identification of essential genes in *Caenorhabditis elegans* through whole-genome sequencing of legacy mutant collections

**DOI:** 10.1093/g3journal/jkab328

**Published:** 2021-09-22

**Authors:** Erica Li-Leger, Richard Feichtinger, Stephane Flibotte, Heinke Holzkamp, Ralf Schnabel, Donald G Moerman

**Affiliations:** 1 Department of Zoology, University of British Columbia, Vancouver, BC V6T 1Z3, Canada; 2 Department of Developmental Genetics, Institute of Genetics, Technische Universität, Braunschweig 38106, Germany; 3 UBC/LSI Bioinformatics Facility, University of British Columbia, Vancouver, BC V6T 1Z3, Canada

**Keywords:** C. elegans, essential genes, maternal-effect, embryogenesis, fertilization, legacy mutants, whole-genome sequencing

## Abstract

It has been estimated that 15%–30% of the ∼20,000 genes in *C. elegans* are essential, yet many of these genes remain to be identified or characterized. With the goal of identifying unknown essential genes, we performed whole-genome sequencing on complementation pairs from legacy collections of maternal-effect lethal and sterile mutants. This approach uncovered maternal genes required for embryonic development and genes with apparent sperm-specific functions. In total, 58 putative essential genes were identified on chromosomes III–V, of which 52 genes are represented by novel alleles in this collection. Of these 52 genes, 19 (40 alleles) were selected for further functional characterization. The terminal phenotypes of embryos were examined, revealing defects in cell division, morphogenesis, and osmotic integrity of the eggshell. Mating assays with wild-type males revealed previously unknown male-expressed genes required for fertilization and embryonic development. The result of this study is a catalog of mutant alleles in essential genes that will serve as a resource to guide further study toward a more complete understanding of this important model organism. As many genes and developmental pathways in *C. elegans* are conserved and essential genes are often linked to human disease, uncovering the function of these genes may also provide insight to further our understanding of human biology.

## Teaser

Estimates suggest that 15-30% of *C. elegans* genes are essential, yet many of these remain to be identified or characterized. The authors performed whole-genome sequencing on legacy collections of maternal-effect lethal and sterile mutants, uncovering novel alleles representing 52 essential genes. 19 genes were selected for further functional characterization, revealing defects in embryogenesis and fertilization. The resulting catalogue of mutant alleles in essential genes is a resource to guide further study toward a more complete understanding of this important model organism.

## Introduction

Essential genes are those required for the survival or reproduction of an organism, and therefore encode elements that are foundational to life. This class of genes has been widely studied for a number of reasons. Essential genes are often well conserved and can offer insight into the principles that govern common biological processes (Hughes 2002; Jordan *et al.* 2002; Georgi *et al.* 2013). Researching these genes and their functions has important implications in understanding the cellular and developmental processes that form complex organisms, including humans. In addition, identifying genes that are lethal when mutated opens up new avenues through which drug development approaches can target parasites, pathogens, and cancer cells (*e.g.*, Doyle *et al.* 2010; Shi *et al.* 2015; Vyas *et al.* 2015; Zhang *et al.* 2018). Finally, the concept of a minimal gene set that is comprised of all genes necessary for life has been the subject of much investigation and has recently been of particular interest in the field of synthetic biology (reviewed in Ausländer *et al.* 2017).

Studying essential genes in humans is complicated by practical and ethical considerations. Accordingly, model organisms have played a key role in identifying and understanding essential genes, and efforts have been made to identify all essential genes in a few model organisms. Systematic genome-wide studies of gene function in *Saccharomyces cerevisiae* have uncovered more than 1100 essential genes, many of which have phylogenetically conserved roles in fundamental biological processes such as cell division, protein synthesis, and metabolism (Winzeler *et al.* 1999; Giaever *et al.* 2002; Yu *et al.* 2006; Li *et al.* 2011). While an important contribution, this is only a fraction of all the essential genes in multicellular organisms. In more complex model organisms, identifying all essential genes in the genome has not been so straightforward. The discovery of RNA interference (RNAi; Fire *et al.* 1998) enabled researchers to employ genome-wide reverse genetic screens to examine the phenotypic effects of gene knockdown (Fraser *et al.* 2000; Kamath *et al.* 2003). In general, this has been an effective, high-throughput method for identifying many genes with essential functions (Gönczy *et al.* 2000; Sönnichsen *et al.* 2005). However, there are limitations to using RNAi to screen for all essential genes, including incomplete gene knock down, off-target effects, and RNAi resistance in a certain tissue or cell types; thus, many genes of biological importance escape identification in high-throughput RNAi screens. This highlights the motivation to obtain null alleles for every gene in the genome, which has been the goal of several model organism consortia (Bradley *et al.* 2012; *C.**elegans* Deletion Mutant Consortium 2012; Varshney *et al.* 2013), though it has not yet been achieved for any metazoan.

*Caenorhabditis elegans* has been an important model in developmental biology for decades, and the ability to freeze and store populations of *C. elegans* indefinitely allows investigators to share their original mutant strains with others around the world. In the first few decades of *C. elegans* research, dozens of forward genetics screens were used to uncover mutants in hundreds of essential genes (*e.g.*, Herman 1978; Meneely and Herman 1979; Rogalski *et al.* 1982; Howell *et al.* 1987; Clark *et al.* 1988; Johnsen and Baillie 1988, 1991; Kemphues *et al.* 1988; McKim *et al.* 1988, 1992; Howell and Rose 1990; Stewart *et al.* 1998; Gönczy *et al.* 1999). These early studies generated what we refer to here as legacy collections. The alleles were often mapped to a region of the genome through deficiency or linkage mapping. However, the process of identifying the molecular nature of the genetic mutations one-by-one using traditional methods was slow and laborious before the genome sequence was complete (The *C. elegans* Sequencing Consortium 1998) and next-generation sequencing technologies were developed (reviewed in Metzker 2010; Goodwin *et al.* 2016).

As whole-genome sequencing (WGS) has become widely adopted, methods for identifying mutant alleles have evolved to take advantage of these technological advances (Sarin *et al.* 2008; Smith *et al.* 2008, 2016; Srivatsan *et al.* 2008; Blumenstiel *et al.* 2009; Schneeberger *et al.* 2009; Doitsidou *et al.* 2010; Flibotte *et al.* 2010; Zuryn *et al.* 2010). With WGS becoming increasingly affordable over time, mutant collections can now be mined for data in efficient ways that were not possible two decades ago. Performing WGS on a single mutant genome is often insufficient to identify a causal variant due to the abundance of background mutations in any given strain, particularly one that has been subjected to random mutagenesis (Denver *et al.* 2004; Hillier *et al.* 2008; Sarin *et al.* 2008; Flibotte *et al.* 2010). However, when paired with additional strategies such as deletion or SNP-based mapping or bulk segregant analysis, WGS becomes a valuable tool to expedite gene identification. Furthermore, if multiple independently derived allelic mutants exist, an even simpler approach can be taken. By sequencing two or more mutants within a complementation group and looking for mutations in the same gene, the need for additional mapping or crossing schemes is greatly reduced (Schneeberger and Weigel 2011; Nordström *et al.* 2013).

In the legacy mutant collections described above, where large numbers of mutants are isolated, it is feasible to obtain complementation groups with multiple alleles for many loci. In addition, the abundance of mutants obtained in these large-scale genetic screens suggests that some legacy mutant collections may harbor strains for which the mutations remain unidentified. If such collections are coupled with thorough annotations, they are valuable resources that can be mined with WGS. Indeed, some investigators have recently used such WGS-based approaches to uncover novel essential genes from legacy collections (Jaramillo-Lambert *et al.* 2015; Qin *et al.* 2018). These projects bring us closer to identifying all essential genes in *C. elegans* and also contribute to the ongoing efforts to obtain null mutations in every gene in the genome.

There are currently 3755 *C. elegans* genes that have been annotated with lethal or sterile phenotypes from RNAi knockdown studies (data from WormBase version WS275). In comparison, the number of genes currently represented by lethal or sterile mutant alleles is 1885 (data from WormBase version WS275). These numbers should be considered minimums, as the database annotations are not necessarily up to date. The discrepancy in these numbers could be illustrative of the comparatively time-consuming and laborious nature of isolating and identifying mutants. In addition, some of the genes identified as essential in RNAi screens may belong to paralogous gene families whose redundant functions are masked in single gene knockouts. Although the total number of essential genes in *C. elegans* is unknown, extrapolation from saturation mutagenesis screens has led to estimates that approximately 15%–30% of the ∼20,000 genes in this organism are essential (Clark *et al.* 1988; Howell and Rose 1990; Johnsen and Baillie 1997; The *C.**elegans* Deletion Mutant Consortium 2012). This suggests the possibility that there are many essential genes in *C. elegans* that remain unidentified and/or lack representation by a null allele.

In this study, we use WGS to revisit two *C. elegans* legacy mutant collections isolated more than 25 years ago. These collections are a rich resource for essential gene discovery; they comprise 75 complementation groups in which at least two alleles with sterile or maternal-effect lethal phenotypes have been found. With these collections, we sought to identify novel essential genes and to conduct a preliminary characterization of their roles in fertilization and development. Wild-type male rescue assays are used to attribute some mutant phenotypes to sperm-specific genetic defects. In addition, we examine arrested embryos using differential interference contrast (DIC) microscopy and document their terminal phenotypes. This work comprises a catalog of 125 alleles with mutations in 58 putative essential genes on chromosomes III–V. Of these 58 genes, 52 are represented by novel alleles in this collection. We present several genes which are reported here for the first time as essential genes and mutant alleles for genes that have only previously been studied with RNAi knockdown. This work aims to help accelerate research efforts by identifying essential genes and providing an entry point into further investigations of gene function. Advancing our understanding of essential genes is imperative to reaching a more comprehensive knowledge of gene function in *C. elegans* and may provide insight into conserved processes in developmental biology, parasitic nematology, and human disease.

## Materials and methods

### Generation of legacy mutant collections

Mutant strains were isolated in screens for maternal-effect lethal and sterile alleles in the early 1990s by Heinke Holzkamp and Ralf Schnabel (unpublished data), and Richard Feichtinger (Feichtinger 1995). Two balancer strains were used for mutagenesis; GE1532: *unc-32(e189)/qC1 [dpy-19(1259) glp-1(q339)] III; him-3(e1147) IV* and GE1550: *him-9(e1487) II; unc-24(e138)/nT1[let(m435)] IV; dpy-11(e224)/nT1[let(m435)] V.* These parental strains were subjected to ethyl methanesulfonate (EMS) mutagenesis at 20° as described by Brenner (1974), with a mutagen dose of 50–75 mM and duration between 4 and 6 h. Following mutagenesis, L4 F1 animals were singled on plates at either 15° or 17°. Animals with homozygous markers in the F2 or F3 generation were transferred to 25° and subsequently screened for the production of dead eggs, unfertilized oocytes, or no eggs laid. The two mutant collections analyzed in this study are summarized in Table 1.

**Table 1:** Summary of mutant collections

Collection	Number of complementation groups with ≥2 alleles	Chromosome	Mutant genotypes
A	32	III	*unc-32(e189) let(t…)/qC1 III; him-3(e1147) IV*
B	25	IV	*him-9(e1487) II; unc-24(e138) let(t…)/nT1 [let(m435)] IV; dpy-11(e224)/nT1 [let(m435)] V*
	18	V	*him-9(e1487) II; unc-24(e138)/nT1 [let(m435)] IV; dpy-11(e224) let(t…)/nT1 [let(m435)] V*

### List of strains

The wild-type Bristol N2 derivative PD1074 and strains with the following mutations were used: *him-3(e1147), unc-32(e189), qC1[dpy-19(e1259) glp-1(q339)]*, *him-9(e1487), unc-24(e138), dpy-11(e224, e1180), nT1[let(m435)] (IV; V), nT1[unc(n754)let] (IV; V)*. Strains carrying the following deletions were used for deficiency mapping: *nDf16, nDf40, sDf110, sDf125, tDf5, tDf6, tDf7 (III)*; *eDf19, nDf41, sDf2, sDf21, stDf7 (IV); ctDf1, itDf2, nDf32, sDf28, sDf35 (V)*. All *sDfs* were kindly provided by D. Baillie's Lab (Simon Fraser University), and some strains were kindly provided by the Caenorhabditis Genetics Center (University of Minnesota). Nematode strains were cultured as previously described by Brenner (1974).

### Outcrossing, mapping, and complementation analysis

All mutant strains were outcrossed at least once to minimize background mutations on other chromosomes. Hermaphrodites of the mutant strains were outcrossed with males of GE1532 [*unc-32(e189)/qC1 [dpy-19(1259) glp-1(q339)] III; him-3(e1147) IV*] for Collection A and males of GE1964: *him-9(e1487) II; +/nT1[let(m435)] IV; dpy-11(e1180)/nT1[let(m435)] V* for Collection B. Deficiency mapping was used to localize mutations to a chromosomal region using the deletion strains listed above. A detailed description of the outcrossing and mapping schemes for Collection B can be found in Supplementary File S1 and Feichtinger (1995).

Complementation analysis of legacy mutants was performed by crossing 10 males of one mutant strain to 4 hermaphrodites of another strain. The presence of males with homozygous markers indicated successful crossing, and homozygous hermaphrodite progeny were transferred to new plates to determine whether viable offspring were produced and thus complementation occurred. Failure to complement was verified with additional homozygous animals or by repeating the cross. Complementation tests between CRISPR-Cas9 deletion strains and legacy mutants were performed by crossing heterozygous CRISPR-Cas9 deletion (GFP/+) males to heterozygous legacy mutant hermaphrodites. Twenty GFP hermaphrodite F1s were singled on new plates and those segregating viable Dpy and/or Unc progeny indicated complementation between the two alleles.

### DNA extraction

Balanced heterozygous strains were grown on 100 mm nematode growth medium (NGM) agar plates (standard recipe with 3 times concentration of peptone) seeded with OP50 and harvested at starvation. Genomic DNA was extracted using a standard isopropanol precipitation technique previously described (Au *et al.* 2019). DNA quality was assessed with a NanoDrop 2000c Spectrophotometer (Thermo Scientific) and DNA concentration was measured using a Qubit 2.0 Fluorometer and dsDNA Broad Range Assay kit (Life Technologies).

### Whole-genome sequencing and analysis pipeline

DNA library preparation and WGS were carried out by The Centre for Applied Genomics (The Hospital for Sick Children, Toronto, Canada). Between 20 and 33 *C. elegans* mutant strains were run together on one lane of an Illumina HiSeq X to generate 150-bp paired-end reads.

Sequencing analysis was done using a modified version of a previously designed custom pipeline (Flibotte *et al.* 2010; Thompson *et al.* 2013). Reads were aligned to the *C. elegans* reference genome (WS263; wormbase.org) using the short-read aligner BWA version 0.7.16 (Li and Durbin 2009). Single nucleotide variants (SNVs) and small insertions or deletions (indels) were called using SAMtools toolbox version 1.6 (Li *et al.* 2009). To eliminate unreliable calls, variants at genomic locations for which the canonical N2 strain has historically had low read depth or poor quality (Thompson *et al.* 2013) were removed as potential candidates. The variant calls were annotated with a custom Perl script and labeled heterozygous if represented by 20%–80% of the reads at that location. The remaining candidates were then subjected to a series of custom filters: (i) any variants that appeared in more than three strains from the same collection were removed; (ii) homozygous mutations were removed; (iii) only mutations affecting coding exons (indels, missense, and nonsense mutations) or splice sites (defined as the first two and last two base pairs in an intron) were kept, while all variants from other noncoding regions were removed; and (iv) only mutations on the chromosome to which the mutation had originally been mapped were selected, while variants on all other chromosomes were removed.

For each pair of strains belonging to a complementation group, the final list of candidate mutations was compared and the gene or genes in common were identified. In cases where there was only one gene in common on both lists, this gene was designated the putative essential gene. For complementation groups with multiple candidate genes in common, additional information such as the nature of the mutations and existing knowledge about the genes was used to select a single candidate gene, when possible. When there was no gene candidate in common within a pair of strains, the list of variants was reanalyzed to look for larger deletions and rearrangements. If available, two additional alleles were sequenced to help identify the gene.

### Validation of gene candidates

To validate the candidate gene candidates derived from WGS analysis, the genomic position of each candidate gene was corroborated with the legacy data from deficiency mapping experiments. Approximate boundaries for the deletions were estimated from the map coordinates of genes known to lie internal or external to the deletions according to data from WormBase (WS275).

For further validation of select gene candidates, deletion mutants were generated in an N2 wild-type background using a CRISPR-Cas9 genome editing strategy previously described (Norris *et al.* 2015; Au *et al.* 2019). Two guide RNAs were used to excise the gene of interest and replace it with a selection cassette expressing G418 drug resistance and pharyngeal GFP (*loxP* + P*myo-2*::*GFP*::*unc-54* 3′UTR + P*rps-27*::*neoR*::*unc-54*3′UTR *+ loxP* vector, provided by Dr. John Calarco, University of Toronto, Canada). Guide RNAs were designed using the *C. elegans* Guide Selection Tool (genome.sfu.ca/crispr) and synthesized by Integrated DNA Technologies (IDT). Repair templates were generated by assembling homology arms (450-bp gBlocks synthesized by IDT) and the selection cassette using the NEBuilder Hifi DNA Assembly Kit (New England Biolabs).

Cas9 protein (generously gifted from Dr. Geraldine Seydoux) was assembled into a ribonucleoprotein (RNP) complex with the guide RNAs and tracrRNA (IDT) following the manufacturer’s recommendations. PD1074 animals were injected using standard microinjection techniques (Mello *et al.* 1991; Kadandale *et al.* 2009) with an injection mix consisting of: 50 ng/µl repair template, 0.5 µM RNP complex, 5 ng/µl pCFJ104 (P*myo-3*::mCherry), and 2.5 ng/µl pCFJ90 (P*myo-2*::mCherry). Injected animals were screened according to the protocol described in Norris *et al.* (2015) and genomic edits were validated using the PCR protocol described in Au *et al.* (2019). Complementation tests between CRISPR-Cas9 alleles and legacy mutant alleles were performed to verify gene identities, as described above.

### Analysis of orthologs, GO, and expression patterns

Previously reported phenotypes from RNAi experiments or mutant alleles were retrieved from WormBase (WS275) and GExplore (genome.sfu.ca/gexplore; Hutter *et al.* 2009; Hutter and Suh 2016). Life stage-specific gene expression data from the modENCODE project (Hillier *et al.* 2009; Gerstein *et al.* 2010, 2014; Boeck *et al.* 2016) were also accessed through GExplore. Visual inspection of these data revealed genes with maternal expression patterns (high levels of expression in the early embryo and hermaphrodite gonad) as well as those predominantly expressed in males.

Human orthologs of *C. elegans* genes were determined using Ortholist 2 (ortholist.shaye-lab.org; Kim *et al.* 2018). For maximum sensitivity, the minimum number of programs predicting a given ortholog was set to one. For genes with no human orthologs, NCBI BLASTp (blast.ncbi.nlm.nih.gov; Altschul *et al.* 1990) was used to examine distributions of homologs across species and potential nematode-specificity. Protein sequences from the longest transcript of each gene were used to query the nonredundant protein sequences (nr) database, with default parameters and a maximum of 1000 target sequences. The results were filtered with an *E*-value threshold of 10^−5^.

Gene ontology (GO) term analysis was performed using PANTHER version 16.0 (Thomas *et al.* 2003). The list of 58 candidate genes was used for an overrepresentation test, with the set of all *C. elegans* genes as a background list. Overrepresentation was analyzed with a Fisher’s Exact test and *P*-values were adjusted with the Bonferroni multiple testing correction.

### Temperature sensitivity and mating assays

To assay temperature sensitivity, heterozygous strains were propagated at 15° and homozygous L4 animals were isolated on 60 mm NGM plates (2 × 6/plate or 3 × 3/plate). After 1 week at 15°, plates were screened for the presence of viable homozygous progeny. If present, L4 homozygotes were transferred to new plates at 25° and screened after 3 days to confirm lethality or sterility.

Mating assays were carried out using PD1074 males and mutant hermaphrodites. Three L4-stage homozygous mutant hermaphrodites were isolated and crossed with 10 PD1074 males on each of three 60 mm NGM plates. Control plates consisted of three L4 hermaphrodite mutants without males. Mating assays were carried out at 25°C and observations were taken after 3 days, noting the absence or presence of viable cross progeny.

### Microscopy

The terminal phenotypes of dead eggs from maternal-effect lethal mutants were observed using DIC microscopy. Young adult homozygous mutants were dissected to release their eggs in either M9 buffer with Triton X-100 (0.5%; M9+TX) or distilled water and embryos were left to develop at 25°C overnight (∼16 h). Embryos were mounted on 2% agarose pads and visualized using a Zeiss Axioplan 2 equipped with DIC optics. Images of representative embryos were captured using a Zeiss Axiocam 105 Color camera and ZEN 2.6 imaging software (Carl Zeiss Microscopy). For embryos incubated in distilled water, an osmotic integrity defective (OID) phenotype was noted for embryos that burst or swelled and filled the eggshell, as described by Sönnichsen *et al.* (2005).

## Results

### Identification of 58 putative essential genes

WGS was performed on a total of 157 strains, with depth of coverage ranging between 21x and 65x (average = 38x). A minimum of two alleles for each of 75 complementation groups were sequenced and a total of 58 putative essential genes were identified (Table 2). Literature searches revealed that 49 of these genes have been annotated with lethal or sterile phenotypes from either mutant alleles or RNAi studies. Furthermore, 46 of the 157 alleles have been previously mentioned in publications with some phenotypic description (Vatcher *et al.* 1998; Gönczy *et al.* 1999, 2001; Molin *et al.* 1999; Kaitna *et al.* 2002; Brauchle *et al.* 2003; Cockell *et al.* 2004; Delattre *et al.* 2004; Sonneville and Gönczy 2004; Bischoff and Schnabel 2006; Langenhan *et al.* 2009; Nieto *et al.* 2010; von Tobel *et al.* 2014). Although 18 of these alleles have been previously sequenced, we were unaware of this when initially analyzing the data, and these alleles therefore served as a blind test set to validate our analysis approach. Eight of the nine genes represented in this set of 18 previously sequenced alleles were correctly identified by our pipeline. The gene *cul-2* (Sonneville and Gönczy 2004) escaped identification due to an intronic mutation in one allele that did not pass our filtering criteria but was found upon manual inspection of the sequencing data. A complete list of previously published and sequenced alleles can be found in Supplementary File S2 with their associated publications.

**Table 2:** List of 58 putative essential genes with associated maternal-effect lethal or sterile alleles

Legacy comp. groupa	Strain	Allele(s)	Gene	Chr.	Position	Base change		Mutation	Mutation type	Amino acid changeb	Protein size (Amino Acids)b	Human ortholog(s)	Associated OMIM phenotype(s)c
Y	GE2430	t2135	*air-1*	V	8221773	C	T	SNV	Missense	R62C	326	AURKA, AURKB, AURKC, STK36	Colorectal cancer, susceptibility to [114500]; Spermatogenic failure 5 [243060]
	GE2337	t2095	*air-1*	V	8223169	CAT	C	Deletion	Frameshift	—			
x	GE2314	t1724	*aptf-2*	IV	13414105	A	G	SNV	Missense	L244P	367	TFAP2A, TFAP2B, TFAP2C, TFAP2D, TFAP2E	Char syndrome [169100]; Patent ductus arteriosus 2 [617035]; Branchiooculofacial syndrome [113620]
	GE2289	t1836	*aptf-2*	IV	13414263	G	T	SNV	Nonsense	C191*			
H	GE1958	t1726	*atg-7*	IV	11079764	G	A	SNV	Nonsense	Q367*	647	ATG7	None
	GE1936	t1738	*atg-7*	IV	11079973	C	T	SNV	Nonsense	W311*			
T	GE2449	t2143	*atl-1*	V	9635587	C	T	SNV	Nonsense	W2346*	2531	ATR, PRKDC	Cutaneous telangiectasia and cancer syndrome, familial [614564]; Seckel syndrome 1 [210600]; Immunodeficiency 26 with or without neurologic abnormalities [615966]
	GE2467	t2155	*atl-1*	V	9637978	C	T	SNV	Missense	E1710K			
gene-28	GE2200	t1480	*bckd-1A*	III	12969933	G	A	SNV	Nonsense	Q174*	432	BCKDHA, TMEM91, AC011462.1	Maple syrup urine disease [248600]
	GE1742	t1461	*bckd-1A*	III	12971429	G	A	SNV	Nonsense	Q109*			
gene-17	GE2206	t1514	*bckd-1A*	III	12971273	G	A	SNV	Nonsense	Q161*			
	GE2627	t1603	*bckd-1A*	III	12971305	C	T	SNV	Nonsense	W150*			
vz	GE2890	t1821	*C34D4.4*	IV	7150054	G	A	SNV	Nonsense	W101*	205	TVP23A, TVP23B, TVP23C, TVP23C-CDRT4	None
	GE2840	t1860	*C34D4.4*	IV	7150143	G	A	SNV	Nonsense	W131*			
a	GE2734	t2029	*C56A3.8*	V	13560728	G	A	SNV	Missense	G62E	402	PI4K2A, PI4K2B	None
	GE2886	t2055	*C56A3.8*	V	13560787	G	A	SNV	Missense	E243K			
	GE2487	t2149	*C56A3.8*	V	13561369	C	T	SNV	Missense	P82L			
V	GE2142	t2074	*ccz-1*	V	13679756	T	A	SNV	Nonsense	Y248*	528	CCZ1, CCZ1B	None
	GE2304	t2129^d^	*ccz-1*	V	13680792	C	T	SNV	Nonsense	Q361*			
b	GE2047	t2021	*cept-2*	V	14349388	G	A	SNV	Nonsense	W128*	424	CEPT1, CHPT1, SELENOI	Spastic paraplegia 81, autosomal recessive [618768]
	GE2122	t2007	*cept-2*	V	14349747	G	A	SNV	Splice site	—			
gene-4	GE2275	t1517	*cls-2*	III	9055405	G	A	SNV	Missense	R102Q	1023	CLASP1, CLASP2	None
	GE2357	t1527	*cls-2*	III	9055440	G	A	SNV	Missense	G114R			
R	GE2082	t2053	*cpl-1*	V	16593886	G	A	SNV	Missense	S148F	337	CTSF, CTSK, CTSL, CTSS, CTSV	Pycnodysostosis [265800]; Ceroid lipofuscinosis, neuronal, 13 [615362]
	GE2451	t2144	*cpl-1*	V	16595201	G	A	SNV	Nonsense	Q49*			
A	GE2447	t1879	*cpt-2*	IV	11180120	C	T	SNV	Nonsense	Q141*	646	CPT2	Carnitine palmitoyltransferase II deficiency [600649, 608836, 255110]; Encephalopathy, acute, infection-induced, susceptibility to, 4 [614212]
	GE1938	t1742	*cpt-2*	IV	11180603	G	A	SNV	Nonsense	W194*			
gene-24	GE2657	t1704	*cra-1*	III	6867181	G	A	SNV	Nonsense	Q525*	958	NAA25	None
	GE2242	t1618	*cra-1*	III	6868737	C	T	SNV	Nonsense	W149*			
D	GE1929	t1729	*csr-1*	IV	7960467	T	A	SNV	Missense	N708K	1030	None	None
	GE1929	t1729	*csr-1*	IV	7961246	G	A	SNV	Missense	G922E			
	GE2452	t1897	*csr-1*	IV	7959252	G	A	SNV	Splice site	—			
gene-25	GE2595	t1662 t1718	*cup-5*	III	7585568	C	T	SNV	Nonsense	R263*	668	MCOLN1, MCOLN2, MCOLN3	Mucolipidosis IV [252650]
	GE2355	t1528	*cup-5*	III	7590536	G	A	SNV	Splice site	—			
gene-30	GE2345	t1525^d^	*cyk-3*	III	6020590	C	T	SNV	Nonsense	Q98*	1178	USP15, USP32, USP6	None
	GE2352	t1535^d^	*cyk-3*	III	6022863	G	A	SNV	Nonsense	W723*			
J	GE2499	t1877	*D2096.12*	IV	8363937	C	T	SNV	Nonsense	Q126*	763	None	None
	GE2407	t1906	*D2096.12*	IV	8365654	T	A	SNV	Nonsense	L638*			
O	GE2135	t2043	*dgtr-1*	V	6497335	G	A	SNV	Splice site	—	359	AWAT1, AWAT2, DGAT2, DGAT2L6, MOGAT1, MOGAT2, MOGAT3	None
	GE2063	t2042	*dgtr-1*	V	6498186	G	A	SNV	Missense	G310R			
C	GE2028	t1801	*dif-1*	IV	7552230	A	C	SNV	Nonsense	Y187*	312	SLC25A20	Carnitine-acylcarnitine translocase deficiency [212138]
	GE1932	t1732	*dif-1*	IV	7552641	C	T	SNV	Missense	G75D			
gene-13	GE2612	t1676^d^	*div-1*	III	10245480	G	A	SNV	Nonsense	Q489*	581	POLA2	None
	GE2577	t1642	*div-1*	III	10248544	C	T	SNV	Start ATG	M1I			
d	GE2335	t2056	*dlat-1*	V	14445907	G	A	SNV	Nonsense	Q419*	507	DLAT	Pyruvate dehydrogenase E2 deficiency [245348]
	GE2541	t2035	*dlat-1*	V	14446981	G	A	SNV	Missense	P83L			
u	GE2402	t1940	*F21D5.1*	IV	8727315	C	T	SNV	Missense	A436V	550	PGM3	Immunodeficiency 23 [615816]
	GE2445	t1935	*F21D5.1*	IV	8727668	C	T	SNV	Missense	L539F			
t	GE2837	t1791	*F56D5.2*	IV	9397791	G	A	SNV	Nonsense	Q214*	385	None	None
	GE2881	t1744	*F56D5.2*	IV	9398158	G	A	SNV	Missense	S107F			
gene-26	GE1715	t1436	*gsp-2*	III	7337087	C	T	SNV	Nonsense	R95*	333	PPP1CA, PPP1CB, PPP1CC	Noonan syndrome-like disorder with loose anagen hair 2 [617506]
	GE2360	t1481	*gsp-2*	III	7337383	G	A	SNV	Missense	G174E			
gene-32	GE2545	t1577	*gsr-1*	III	3652401	G	A	SNV	Missense	G335R	473	GSR, TXNRD1, TXNRD2, TXNRD3	Hemolytic anemia due to glutathione reductase deficiency [618660]; Glucocorticoid deficiency 5 [617825]
	GE2644	t1594	*gsr-1*	III	3652407	C	T	SNV	Nonsense	R337*			
gene-31	GE2583	t1654	*hcp-3*	III	9615498	G	A	SNV	Missense	R269C	288	CENPA	None
	GE2692	t1717	*hcp-3*	III	9615555	C	T	SNV	Missense	E250K			
G	GE2455	t1914	*klp-18*	IV	7040335	T	C	SNV	Missense	Y42H	932	KIF15	None
	GE2000	t1795	*klp-18*	IV	7041203	G	A	SNV	Missense	E316K			
gene-6	GE2367	t1563	*klp-19*	III	13306451	A	T	SNV	Missense	L230H	1083	KIF4A, KIF4B	Mental retardation, X-linked 100 [300923]
	GE2367	t1563	*klp-19*	III	13306457	G	A	SNV	Missense	A228V			
	GE2264	t1628	*klp-19*	III	13306872	C	T	SNV	Missense	G90R			
I	GE2003	t1817	*let-99*	IV	12569291	C	T	SNV	Nonsense	Q447*	698	None	None
	GE2514	t1912	*let-99*	IV	12570199	C	T	SNV	Missense	L617F			
gene-22	GE2730	t1550^d^	*lis-1*	III	13375376	C	T	SNV	Nonsense	W92*	404	PAFAH1B1	Lissencephaly 1; Subcortical laminar heterotopia [607432]
	GE2653	t1698^d^	*lis-1*	III	13375401	C	T	SNV	Splice site	—			
z	GE2130	t1765	*mbk-2*	IV	13033086	C	T	SNV	Missense	R533C	817	DYRK2, DYRK3, DYRK4	None
	GE2503	t1888	*mbk-2*	IV	13033644	C	T	SNV	Missense	P701L			
gene-10	GE2740	t1576^d^	*mel-32*	III	6440655	C	T	SNV	Missense	G395R	507	SHMT1, SHMT2	None
	GE1731	t1456^d^	*mel-32*	III	6440831	C	T	SNV	Missense	G336E			
M	GE1999	t1793	*mex-5*	IV	13354014	T	G	SNV	Nonsense	Y79*	468	None	None
	GE2093	t1800	*mex-5*	IV	13354478	T	A	SNV	Nonsense	L219*			
S	GE2511	t2162	*mom-2*	V	8356808	T	G	SNV	Missense	C80G	362	WNT11, WNT9A, WNT9B	None
	GE2523	t2180	*mom-2*	V	8357121	T	C	SNV	Missense	C139R			
W	GE2497	t2137	*mre-11*	V	10735712	G	A	SNV	Missense	H269Y	728	MRE11	Ataxia-telangiectasia-like disorder 1 [604391]
	GE2103	t2092	*mre-11*	V	10736080	A	G	SNV	Missense	F146S			
v	GE2091	t1772	*nstp-2*	IV	6604731	A	T	SNV	Missense	L277H	324	SLC35B4	None
	GE2288	t1835	*nstp-2*	IV	6605266	C	T	SNV	Missense	G131R			
F	GE2391	t1932	*perm-5*	IV	5696931	A	T	SNV	Missense	C454S	518	None	None
	GE2453	t1900	*perm-5*	IV	5698096	A	G	SNV	Missense	S323P			
gene-21	GE2237	t1614	*pod-1*	III	13518266	G	A	SNV	Missense	A912V	1136	CORO7, CORO7-PAM16	None
	GE2605	t1674	*pod-1*	III	13518357	G	A	SNV	Nonsense	R882*			
U	GE3128	t2177	*pos-1*	V	8414544	G	A	SNV	Splice site	—	264	None	None
	GE2101	t2080	*pos-1*	V	8414579	T	A	SNV	Missense	V145D			
Z	GE2517	t2175	*rad-50*	V	12247914	T	A	SNV	Nonsense	L350*	1312	RAD5, AC116366.3	Nijmegen breakage syndrome-like disorder [613078]
	GE2476	t2147	*rad-50*	V	12250324	T	A	SNV	Missense	I1101N			
E	GE2189	t1750	*rad-51*	IV	10282013	A	T	SNV	Missense	I384N	395	DMC1, RAD51, RAD51B, RAD51C, RAD51D	Fanconi anemia, complementation group R, group O [617244, 613390]; Mirror movements 2 [614508]; Breast-ovarian cancer, familial, susceptibility to, 3 [613399]
	GE2433	t1885	*rad-51*	IV	10282328	C	T	SNV	Missense	V323I			
gene-11	GE2347	t1519	*rmd-1*	III	9759805	G	A	SNV	Missense	G89R	226	RMDN2, RMDN3	None
	GE2219	t1501	*rmd-1*	III	9759929	G	A	SNV	Missense	R130H			
gene-18	GE2211	t1476^d^	*sas-1*	III	12710102	C	T	SNV	Missense	P419S	570	None	None
	GE2343	t1521^d^	*sas-1*	III	12710202	G	A	SNV	Missense	G452E			
f	GE2078	t2033^d^	*sas-5*	V	11612449	C	T	SNV	Missense	R397C	404	None	None
	GE2134	t2079^d^	*sas-5*	V	11612449	C	T	SNV	Missense	R397C			
P	GE2469	t2173	*spn-4*	V	6783986	A	T	SNV	Nonsense	L259*	351	RBFOX1, RBFOX2, RBFOX3	None
	GE2317	t2098	*spn-4*	V	6784646	A	T	SNV	Missense	V55D			
g	GE2386	t2165	*sqv-4*	V	10660827	G	A	SNV	Missense	P182L	481	UGDH	Epileptic encephalopathy, early infantile, 84 [618792]
	GE2059	t2025	*sqv-4*	V	10661143	G	A	SNV	Missense	S93L			
gene-5	GE2277	t1496	*such-1*	III	11515520	G	A	SNV	Missense	L686F	798	ANAPC5	None
	GE2277	t1496	*such-1*	III	11515883	G	A	SNV	Missense	H565Y			
	GE2666	t1693	*such-1*	III	11515540	C	T	SNV	Missense	R679K			
q	GE2827	t1786	*T22B11.1*	IV	4692945	G	A	SNV	Nonsense	W35*	468	None	None
	GE2895	t1866	*T22B11.1*	IV	4696017	G	A	SNV	Nonsense	W356*			
gene-12	GE1734	t1438 t1477	*tlk-1*	III	9707175	C	T	SNV	Nonsense	Q412*	965	TLK1, TLK2, TLK2PS1	Mental retardation, autosomal dominant 57 [618050]
	GE2613	t1677	*tlk-1*	III	9708080	G	A	SNV	Missense	A694T			
gene-15	GE2399	t1559	*top-3*	III	11951381	G	A	SNV	Nonsense	Q602*	759	TOP3A	Progressive external ophthalmoplegia with mitochondrial DNA deletions, autosomal recessive 5 [618098]; Microcephaly, growth restriction, and increased sister chromatid exchange 2 [618097]
	GE2220	t1516	*top-3*	III	11958680	C	T	SNV	Missense	G59R			
gene-35	GE1735	t1470	*top-3*	III	11957525	C	T	SNV	Nonsense	W114*			
	GE2958	t1464 t1484	*top-3*	III	11951669	C	T	SNV	Missense	G506R			
L	GE2512	t1909	*trcs-1*	IV	9587541	C	T	SNV	Missense	E373K	428	AADAC, AADACL2, AADACL3, AADACL4, NCEH1	None
	GE1939	t1745	*trcs-1*	IV	9587985	G	A	SNV	Nonsense	Q242*			
c	GE2112	t2037	*unc-112*	V	14692219	C	T	SNV	Missense	R669Q	720	FERMT1, FERMT2, FERMT3	Kindler syndrome [173650]; Leukocyte adhesion deficiency, type III [612840]
	GE2326	t2106	*unc-112*	V	14696546	C	T	SNV	Splice site	—			
gene-27	GE1722	t1435	*vps-33.1*	III	8701605	C	T	SNV	Nonsense	R159*	603	VPS33A, VPS33B, AC048338.1	Mucopolysaccharidosis-plus syndrome [617303]; Arthrogryposis, renal dysfunction [208085]
	GE2366	t1561	*vps-33.1*	III	8702923	G	A	SNV	Nonsense	W536*			
Q	GE2292	t2114	*vps-39*	V	14035713	G	A	SNV	Nonsense	Q754*	926	VPS39	None
	GE1937	t2189	*vps-39*	V	14036143	C	T	SNV	Nonsense	W626*			
	GE2056	t2016	*vps-39*	V	14037839	G	C	SNV	Nonsense	Y122*			
N	GE2153	t1773	*wapl-1*	IV	4444464	C	T	SNV	Nonsense	W348*	748	WAPL	None
	GE2305	t1867	*wapl-1*	IV	4442749-4442872	—	—	122-bp deletion	Deletion	—			
p	GE2738	t1833	*Y54G2A.73*	IV	3000662	A	T	SNV	Nonsense	L341*	380	None	None
	GE2387	t1913	*Y54G2A.73*	IV	3001767	G	A	SNV	Nonsense	R252*			
	GE2884	t1755	*Y54G2A.73*	IV	3008481	C	T	SNV	Splice site	—			
gene-23	GE1713	t1433	*ZK688.9*	III	7882477	C	T	SNV	Nonsense	W135*	281	TIPRL	None
	GE2621	t1587	*ZK688.9*	III	7882717	C	T	SNV	Splice site	—			
gene-14	GE2348	t1518^d^	*zyg-8*	III	12063671	C	T	SNV	Nonsense	R312*	802	DCLK1, DCLK2, DCLK3, DCX	Lissencephaly, X-linked, 1; Subcortical laminar heterotopia, X-linked [300067]
	GE2362	t1547^d^	*zyg-8*	III	12063832	G	A	SNV	Splice site	—			
gene-33	GE1718	t1441^d^	*zyg-8*	III	12069655	A	G	SNV	Missense	D665G			
	GE2533	t1638^d^	*zyg-8*	III	12069369–12069742	—	—	372-bp deletion + 6-bp insertion	Deletion/insertion	—			

a Complementation group determined by complementation analysis of legacy mutants.

b Amino acid position and size derived from the longest transcript (wormbase.org, version WS275).

c Phenotypes retrieved from omim.org.

d Previously sequenced allele.

There were 17 complementation groups that had no common gene candidates in the mapping region after our initial analysis. Three of these allele pairs were later shown to be allelic with other complementation groups and were assigned gene candidates accordingly (see below, Table 4, and Supplementary File S2). We were unable to confidently assign gene candidates for the remaining 14 complementation groups. However, Supplementary File S2 contains the full list of common gene mutations (in both coding and noncoding regions) for each complementation group. This list may be used in conjunction with additional genetic assays to elucidate identities for these genes in the future.

While the list of 58 genes includes many known essential genes, among the known genes are alleles that are novel genetic variants. Nineteen genes from this collection which were not previously studied or were not represented by lethal or sterile mutants were designated genes of interest (GOI; Table 3). These 19 GOI, represented by 40 alleles, were further characterized as part of this study. They include 14 genes (28 alleles) with a maternal-effect lethal phenotype and 5 genes (12 alleles) with a sterile phenotype.

**Table 3:** Genes of interest and associated phenotypes

Strain	Allele	Gene	Protein functiona	**Amino acid** c**hangea**	**RNAi** p**henotypeb**	Mutant phenotype	Embryonic osmotic integrity defect
GE1936	t1738	*atg-7*	E1 ubiquitin-activating-like enzyme orthologous to the autophagic budding yeast protein Apg7p	W311*	Growth variant; dauer body morphology variant; pathogen induced death increased; P granule localization defective; dauer development variant; protein aggregation variant; shortened life span; transgene subcellular localization variant; transgene expression variant; necrotic cell death variant; autophagy variant; antibody staining reduced	Dead embryos	No
GE1958	t1726			Q367*		Dead embryos	No
GE2627	t1603	*bckd-1A*	Predicted mitochondrial protein with alpha-ketoacid dehydrogenase activity	W150*	Shortened life span; small	Dead embryos	Yes
GE2206	t1514			Q161*		Dead embryos	Yes
GE2840	t1860	*C34D4.4*	Predicted to have the following domain: Golgi apparatus membrane protein TVP23-like	W131*	—	Unfertilized oocytes	N/A
GE2890	t1821			W101*		Unfertilized oocytes	N/A
GE2734	t2029	*C56A3.8*	Predicted to have 1-phosphati dylinositol 4-kinase activity	G62E	Larval lethal; accumulated germline cell corpses; germ cell compartment morphology variant; germline nuclear positioning variant; larval arrest; cell membrane organization biogenesis variant; embryonic lethal; rachis narrow; apoptosis variant; maternal sterile; reduced brood size	Unfertilized oocytes	N/A
GE2487	t2149			P82L		Unfertilized oocytes	N/A
GE2886	t2055			E243K		Unfertilized oocytes	N/A
GE2122	t2007	*cept-2*	Predicted to have diacylglycerol cholinephosphotransferase activity and ethanolaminephospho-transferase activity	Splice site	Fat content reduced; embryonic lethal; long	Dead embryos	No
GE2047	t2021			W128*		No eggs laid	Some
						(Dead embryos) [ts]	
GE2275	t1517	*cls-2*	Member of the CLASP family of microtubule-binding proteins	R102Q	Locomotion variant; mitosis variant; univalent meiotic chromosomes; no polar body formation; chromosome segregation variant karyomeres early emb; mitotic chromosome segregation variant; mitotic spindle defective early emb; chromosome segregation variant; embryonic lethal; meiotic spindle defective; meiotic progression during oogenesis variant; exploded through vulva; reduced brood size; antibody subcellular localization variant; meiotic chromosome segregation variant	Dead embryos	N/T
GE2357	t1527			G114R		Dead embryos	No
GE1938	t1742	*cpt-2*	Carnitine palmitoyl transferase	W194*	Embryonic lethal	Dead embryos	No
GE2447	t1879			Q141*		Dead embryos	No
GE2407	t1906	*D2096.12*	—	L638*	Locomotion variant	Dead embryos	Some
GE2499	t1877			Q126*		Dead embryos	Yes
GE2063	t2042	*dgtr-1*	Acyl chain transfer enzyme	G310R	Sterile; sick; oocyte number decreased; germline nuclear positioning variant; oocyte septum formation variant; embryonic lethal; embryo OID early emb; oocyte morphology variant; pachytene region organization variant; reduced brood size; germ cell compartment expansion variant; oogenesis variant	Dead embryos	Some
GE2135	t2043			Splice site		Dead embryos	Yes
GE2541	t2035	*dlat-1*	Predicted to have dihydrolipoyllysine-residue acetyltransferase activity	P83L	Embryonic lethal; slow growth; receptor mediated endocytosis defective; pattern of transgene expression variant; sterile progeny; transgene expression increased; general pace of development defective early emb	Dead embryos	No
GE2335	t2056			Q419*		Dead embryos	No
GE2402	t1940	*F21D5.1*	Predicted to have phosphoacetyl-glucosamine mutase activity	A436V	Sterile; germ cell compartment size variant; rachis wide; rachis morphology variant; accumulated germline cell corpses; germ cell compartment morphology variant; germline nuclear positioning variant; embryonic lethal; embryo OID early emb; apoptosis variant; reduced brood size; oogenesis variant	Dead embryos	Yes
GE2445	t1935			L539F		Dead embryos	Yes
GE2881	t1744	*F56D5.2*	—	S107F	—	Unfertilized oocytes	N/A
GE2837	t1791			Q214*		Unfertilized oocytes	N/A
GE2091	t1772	*nstp-2*	Predicted to have UDP-N-acetylglucosamine and UDP-xylose transmembrane transporter activity	L277H	Lysosome-related organelle morphology variant; transgene subcellular localization variant; RAB-11 recycling endosome localization variant; RAB-11 recycling endosome morphology variant	Dead embryos	No
GE2288	t1835			G131R		Dead embryos	No
GE2391	t1932	*perm-5*	Predicted to have lipid binding activity	C454S	Sterile; apoptosis reduced; oocytes lack nucleus; oocyte number decreased; germ cell compartment morphology variant; germline nuclear positioning variant; germ cell compartment anucleate; oocyte septum formation variant; cell membrane organization biogenesis variant; embryonic lethal; embryo OID early emb; oogenesis variant; diplotene region organization variant	Dead embryos	Yes
GE2453	t1900			S323P		Dead embryos	Yes
GE2827	t1786	*T22B11.1*	—	W35*	—	Unfertilized oocytes [ts]	N/A
GE2895	t1866			W356*		Unfertilized oocytes [ts]	N/A
GE2399	t1559	*top-3*	Exhibits DNA topoisomerase type I (single strand cut, ATP-independent) activity	G59R	Chromosome morphology variant; hermaphrodite germline proliferation variant; antibody staining increased; somatic gonad development variant; gonad degenerate; chromosome instability; germ cell mitosis variant; gonad arm morphology variant; meiosis variant; oocyte morphology variant; nuclear appearance variant; fewer germ cells; oogenesis variant	Dead embryos	No
GE2220	t1516			Q602*		Dead embryos	No
GE2512	t1909	*trcs-1*	Putative arylacetamide deacetylase and microsomal lipase	E373K	Apoptosis reduced; diplotene absent during oogenesis; oocyte number decreased; embryo OID early emb; rachis narrow; chromosome condensation variant; pachytene region organization variant; membrane trafficking variant; pachytene progression during oogenesis variant; apoptosis fails to occur; egg laying variant; germ cell compartment expansion absent; embryonic lethal; cell membrane organization biogenesis variant; no oocytes; germ cell compartment expansion variant	Dead embryos [leaky ts]	Yes
GE1939	t1745			Q242*		No eggs laid (dead embryos) [ts]	Yes
GE2884	t1755	*Y54G2A.73*	—	Splice site	—	Unfertilized oocytes	N/A
GE2387	t1913			R252*		Unfertilized oocytes	N/A
GE2738	t1833			L341*		Unfertilized oocytes	N/A
GE1713	t1433	*ZK688.9*	Predicted to have the following domain: TIP41-like protein (TOR signaling pathway regulator)	W135*	Egg laying variant; locomotion variant	Dead embryos	No
GE2621	t1587			Splice site		Dead embryos	No

[ts], temperature-sensitive; N/A, not applicable; N/T, not tested; —, no information available.

a From WormBase (WS275; wormbase.org); amino acid position derived from the longest transcript.

b Phenotypes retrieved from GExplore (genome.sfu.ca/gexplore).

### Validation of candidate gene assignments

After isolation, the mutant alleles were each localized to a chromosomal region through deficiency mapping. These data were used to corroborate the candidate genes derived from WGS analysis and to resolve complementation groups with more than one initial gene candidate. There were 53 complementation groups with only one common gene candidate when coding and splicing variants were restricted to the mapped region. This was considered to be strong evidence that we correctly identified the essential genes. For the five groups which had more than one gene candidate in the mapped region, the nature of the mutations and existing knowledge about the genes in question were used to select a single candidate (Supplementary File S2).

**Figure 1:**
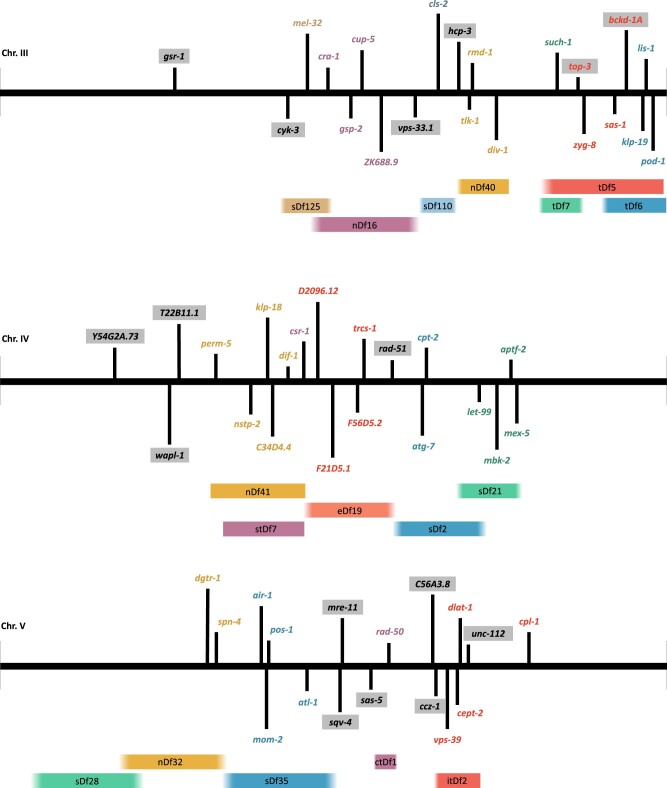
Schematic of gene assignments and deficiency mapping. Genes and deficiencies are shown with their relative positions on chromosomes III–V (coordinates listed in Supplementary File S2). Approximate boundaries of each deficiency were determined by the coordinates of the closest gene known to lie outside of the deletion, when possible (indicated by a faded edge). If no such genes with physical coordinates are known, the outermost gene known to lie inside the deletion was used as the boundary (indicated by a sharp edge). Gene names are colored according to the deficiency under which the alleles were mapped. Genes names assigned to alleles that did not map under any of the tested deficiencies are highlighted in gray. *top-3* and *bckd-1A* on chromosome III are represented by multiple complementation groups with conflicting results from deficiency mapping.

For the majority of complementation groups, the genomic position of the assigned gene is in agreement with the deficiency genetic mapping data (Figure 1). However, with limited information available, it was not possible to assign precise map coordinates to the molecular lesions of the deficiency strains which were used for mapping. For three complementation groups, there is an apparent conflict between the deficiency mapping data and the gene candidates proposed through our analysis. These complementation groups were found to not map under any of the tested deficiencies, but were assigned gene candidates whose genomic coordinates fall into regions covered by the tested deficiencies (alleles of *bckd-1A*, *top-3*, and *unc-112;*Figure 1). In addition, two of these groups were assigned the same gene candidate as another, purportedly distinct, complementation group (Table 4). From WGS analysis, *bckd-1A* was the initial gene candidate for two different complementation groups, yet only one of these groups had been mapped to a deletion (*tDf5*) that covers the *bckd-1A* locus. Similarly, *top-3* was the assigned gene candidate for three different complementation groups, only one of which was mapped under a deficiency (*tDf5*) encompassing that gene. By performing complementation tests with select alleles (Table 4), we concluded that the two *bckd-1A* groups are not distinct, and indeed they contain mutations in the same gene. One of the groups (gene-35) originally identified as *top-3* is a double mutant which fails to complement gene-15 (*top-3*) and gene-34 (unknown gene).

**Table 4:** Complementation tests for conflicting groups

Legacy complementation group	Strain	Allele	Preliminary gene candidate	Mapped under	Complementation test results	Final gene assignment
gene-28	GE1742	t1461	*bckd-1A*	None of tested deficiencies	Fails to complement: GE2206, GE2627	***bckd-1A***
gene-17	GE2627	t1603	*bckd-1A*	*tDf5*	Fails to complement: GE2206, GE1742	***bckd-1A***
	GE2206	t1514		*tDf5*	Fails to complement: GE2627, GE1742	
gene-15	GE2220	t1516	*top-3*	*tDf5*	Fails to complement: GE2399, GE1735	***top-3***
					Complements: GE2278	
	GE2399	t1559		*tDf5*	Fails to complement: GE2220	
gene-34	GE2278	t1502	*top-3*	None of tested deficiencies	Fails to complement: GE1735	**unknown gene**
					Complements: GE2220	
gene-35	GE1735	t1470	*top-3*	None of tested deficiencies	Fails to complement: GE2278, GE2220	**double mutant: *top-3 *+ * *unknown gene**

Three candidate genes (*nstp-2, C34D4.4*, and *F56D5.2*) were selected for additional validation by generating a deletion of the gene in a wild-type background using CRISPR-Cas9 genome editing (Norris *et al.* 2015; Au *et al.* 2019). These genes were chosen because they were expected to be of interest to the broader research community. The deletion alleles have been verified with the PCR protocol described by Au *et al.* (2019). Guide RNA sequences and deletion-flanking sequences are listed in Supplementary File S3. Complementation testing between the newly generated CRISPR-Cas9 deletion mutants and the legacy mutant strains confirmed that the mutations are allelic, and the genes assigned to the legacy strains are correct (Supplementary File S3).

### Human orthologs, gene ontology, and expression patterns

Of the 58 essential gene candidates, 47 genes have predicted human orthologs (Table 2). Many of these genes in humans have been implicated in disease and are associated with OMIM disease phenotypes (Online Mendelian Inheritance in Man; omim.org). BLASTp searches revealed that the set of 19 GOI contains three nematode-specific genes (*F56D5.2*, *perm-5*, and *T22B11.1*) that have homologs in parasitic species, and two uncharacterized genes (*D2096.12* and *Y54G2A.73*) that do not have homology outside the *Caenorhabditis* genus.

**Figure 2:**
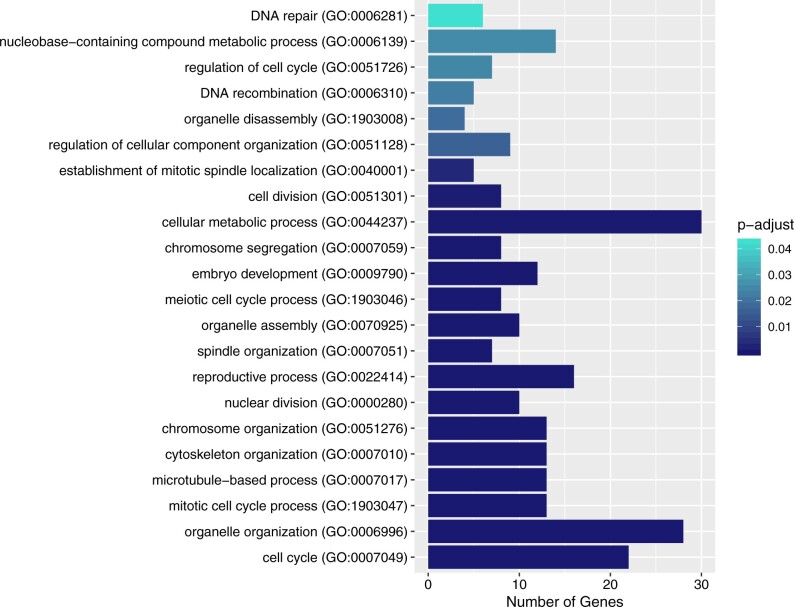
Biological process GO terms overrepresented in the set of 58 putative essential genes. Bar length represents the number of genes in the set associated with each GO term. Overrepresentation was analyzed using PANTHER version 16.0 (Thomas *et al.* 2003) and *P*-values were adjusted with the Bonferroni multiple testing correction. Results were filtered to include terms with adjusted *P* < 0.05 and edited to exclude redundant terms. A list of overrepresented GO terms and associated genes can be found in Supplementary File S3.

To gain insight into the functions of the putative essential genes, an overrepresentation test was used to elucidate the most prominent GO terms associated with them. The biological process terms overrepresented in the set of 58 genes include such terms as organelle organization (GO:0006996), nuclear division (GO:0000280), cellular metabolic process (GO:0044237), and DNA repair (GO:0006281), as shown in Figure 2. In the molecular function category, binding (GO:0005488) and catalytic activity (GO:0003824) are overrepresented by 41 genes (adjusted *P* = 1.2E-07) and 28 genes (adjusted *P* = 1.8E-03), respectively. A complete list of overrepresented GO terms and associated genes can be found in Supplementary File S3.

To examine the timing of gene expression throughout the life cycle, gene expression data from the modENCODE project (Hillier *et al.* 2009; Gerstein *et al.* 2010, 2014; Boeck *et al.* 2016) was retrieved from GExplore (genome.sfu.ca/gexplore; Hutter *et al.* 2009; Hutter and Suh 2016) for the 19 GOI (Supplementary File S4). For 10 of the GOI, these data show high levels of gene expression in the early embryonic stages as well as in adulthood, and particularly in the hermaphrodite gonad. This expression pattern is characteristic of a maternal-effect gene, for which gene products are passed on to the embryo from the parent. Five genes have a maternal gene expression pattern as well as expression throughout other stages of the life cycle, indicating an additional, zygotic role for the gene. Seven genes have elevated expression levels in males and L4-stage hermaphrodites. These genes are suspected to be involved in sperm production or fertilization, and the associated strains were subjected to mating assays (see below).

### Temperature sensitivity and mating assays for genes of interest

The 40 alleles associated with the 19 GOI were further examined to gain insight into the phenotypic consequences of their mutations. Each allele was assayed for temperature sensitivity, as some of the original mutant screening was carried out at 25°C. Five alleles (marked with a [ts] phenotype in Table 3) were deemed temperature-sensitive and could proliferate as homozygotes at a permissive temperature of 15°C, while being maternal-effect lethal or sterile at a restrictive temperature of 25°C. Curiously, four of these temperature-sensitive alleles were the results of stop codons, not missense mutations.

Seven candidate genes (16 alleles) were hypothesized to be involved in male fertility, based on the production of unfertilized oocytes by hermaphrodites and/or predominantly male gene expression patterns. These 16 strains were assayed for their ability to be rescued through mating with wild-type males. 14 of the strains were rescued by the mating assay, while two strains failed to rescue (Table 5). Phenotypic rescue through mating was consistent among alleles of the same gene in five of the seven genes, while two genes had conflicting results among the pair of alleles in their complementation groups (*F56D5.2* and *nstp-2*).

**Table 5:** Putative male fertility genes

Strain	Allele	Gene	Observed mutant phenotype	Successful WT male rescue
GE2627	t1603	*bckd-1A*	Dead embryos	Yes
GE2206	t1514		Dead embryos	Yes
GE2840	t1860	*C34D4.4*	Unfertilized oocytes	Yes
GE2890	t1821		Unfertilized oocytes	Yes
GE2734	t2029	*C56A3.8*	Unfertilized oocytes	Yes
GE2487	t2149		Unfertilized oocytes	Yes
GE2886	t2055		Unfertilized oocytes	Yes
GE2881	t1744	*F56D5.2*	Unfertilized oocytes	No
GE2837	t1791		Unfertilized oocytes	Yes
GE2091	t1772	*nstp-2*	Dead embryos	No
GE2288	t1835		Dead embryos	Yes
GE2827	t1786	*T22B11.1*	Unfertilized oocytes [ts]	Yes
GE2895	t1866		Unfertilized oocytes [ts]	Yes
GE2884	t1755	*Y54G2A.73*	Unfertilized oocytes	Yes
GE2387	t1913		Unfertilized oocytes	Yes
GE2738	t1833		Unfertilized oocytes	Yes

[ts], temperature-sensitive.

### Terminal phenotypes of maternal-effect lethal embryos

**Figure 3:**
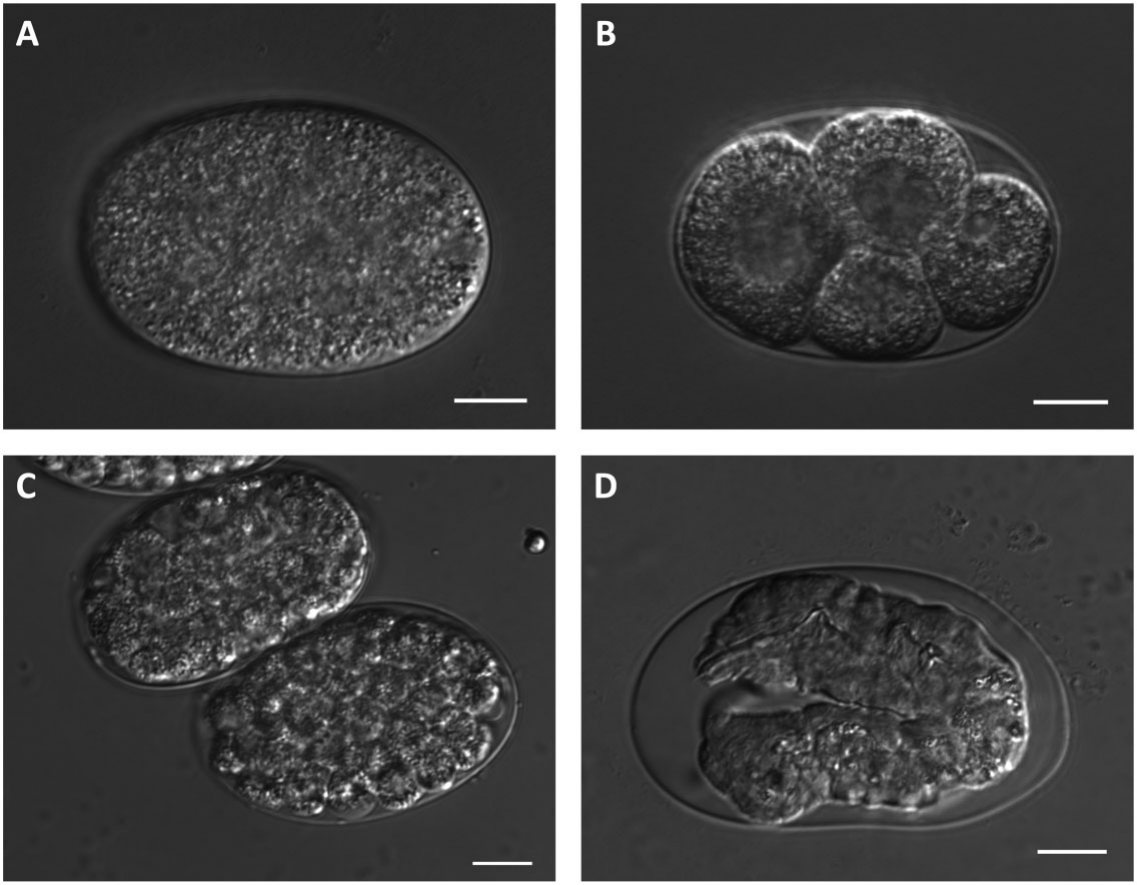
Embryonic arrest visualized with DIC microscopy for select maternal-effect lethal mutants. Eggs were dissected from homozygous mutants and imaged immediately (A) or incubated in distilled water overnight before imaging (B–D). (A) Eggs dissected from *dgtr-1*(*t2043*) homozygotes exhibit signs of an osmotic integrity defect, by filling the eggshell completely. (B) *dlat-1*(*t2035*) embryos exhibit early embryonic arrest, with most embryos consisting of four cells or less. (C) *ZK688.*9(*t1433*) embryos arrest with approximately 100 cells. (D) Terminal embryos of *nstp-2*(*t1835*) have a lumpy body wall morphology and constricted nose; most animals were moving inside the eggshell but did not hatch. All scale bars represent 10 μm.

Using DIC microscopy, the terminal phenotypes of 28 maternal-effect lethal strains (a subset of the 40 GOI strains) were observed. Representative images were selected and compiled into a catalog of terminal phenotypes (Supplementary File S5). Ten strains showed an OID phenotype (as described in Sönnichsen *et al.* 2005) in nearly all embryos after incubation in distilled water, while three additional strains had only some embryos that exhibited this phenotype (Table 3). The OID phenotype was evident in embryos that filled the eggshell completely [for example, *dgtr-1*(*t2043*), Figure 3A] and eggs that burst in their hypotonic surroundings. Early embryonic arrest was observed in embryos from the two *dlat-1* mutant alleles (*t2035* and *t2056*), which arrested most often with only one to four cells (*e.g.*, Figure 3B). Eleven strains had embryos that terminated with approximately 100–200 cells [for example, *ZK688.*9(*t1433*), Figure 3C]; while four strains developed into two- or threefold stage embryos that did not hatch and exhibited clear morphological defects, such as *nstp-2*(*t1835*) with a lumpy body wall and constricted nose tip (Figure 3D).

## Discussion

### Revisiting legacy mutant collections with WGS

In this study, we focused on reexamining legacy collections of *C. elegans* mutants isolated before the complete genome sequence was published (The *C. elegans* Sequencing Consortium 1998) and long before massively parallel sequencing was widely available. With major advances in sequencing technology in the past 30 years (reviewed in Goodwin *et al.* 2016), WGS has become affordable and accessible, making it possible to revisit past projects with new approaches and advanced capabilities. We have sequenced paired alleles from 75 complementation groups on chromosomes III–V, from which we identified 58 putative essential genes (Table 2).

While WGS is a powerful tool, it does not stand alone as a solution to identifying mutant alleles. This study has shown the power of having multiple alleles in a complementation group when faced with the abundance of genomic variants found in WGS analysis. Indeed, when we sequenced four single alleles, which had no complementation pairs, we were unable to designate a single mutation as the variant responsible for maternal-effect lethality (data not shown). Our approach to gene identification proved to be effective and was validated by a combination of different methods. The blind test set of 18 previously sequenced alleles from which eight of nine genes were readily identified serves as an important validation of our analysis pipeline and gives confidence in the results we obtained. In addition, the deficiency mapping data, gene expression patterns from the modENCODE project, GO term analysis, and phenotypes documented from previous experiments provide evidence to support the gene candidates we assigned in these mutant collections.

The CRISPR-Cas9 deletion alleles we generated for selected gene candidates provide additional validation and will be made available to the research community to serve as useful tools for future studies. While the mutant alleles from the original study have been outcrossed, the genetic balancer background and additional mutations that persist can complicate phenotypic analysis. In contrast, these new CRISPR-Cas9 deletion strains were made in a wild-type background, which makes it much easier to handle them and interpret their mutant phenotypes. Furthermore, the pharyngeal GFP expression introduced by the gene-editing approach acts as a dominant and straightforward marker for tracking the alleles in a heterozygous population. This is useful, as the homozygous animals do not produce viable progeny.

The complementation groups that could not be assigned gene candidates in our analysis may have been complicated by variants in noncoding regions, poor sequencing coverage, or inaccurate complementation pairing, among other possibilities. In future work, tracking down the genes we were unable to identify will require repeating complementation tests and re-tooling the analysis approach.

Among the 19 GOI are four temperature-sensitive alleles, all the result of nonsense mutations. While unusual, temperature-sensitive nonsense alleles are not unprecedented and have been found in several organisms (*e.g.*, Golden and Riddle 1984; Samson *et al.* 1995). Both nonsense alleles of *T22B11.1* are temperature-sensitive. This makes us suspicious that perhaps the wild-type process this gene is involved in is itself temperature-dependent. This idea stems from the observation that all alleles of the dauer constitutive genes *daf-4* and *daf-7* are temperature-sensitive (Golden and Riddle 1984). These genes have both amber stop alleles and missense alleles and all are temperature-sensitive. If *T22B11.1* were indeed involved in a temperature-dependent process we would expect a deletion allele to also be temperature-sensitive. The gene *trcs-1* also has a temperature-sensitive nonsense allele and, in addition, it has a leaky temperature-sensitive missense allele. Again, the product of this gene may be involved in a temperature-dependent process. One requires a different explanation for *cept-2* where we have identified two alleles and only one, the nonsense allele, is temperature-sensitive. In *Drosophila*, the *elav* gene has temperature-sensitive alleles that are nonsense alleles and yet they make full-length proteins (Samson *et al.* 1995). A detailed study of this gene and its gene product concluded that, at some low level, an alternative amino acid is substituted at the stop site, allowing for a full length but unstable protein (Samson *et al.* 1995). This type of information suppression suggests we may observe a low-abundance, full-length protein product for *cept-2*.

### GO analysis reveals common themes and gaps in our knowledge

The underlying biological themes of the 58 putative essential genes were revealed by examining their GO terms. The biological processes represented in Figure 2 help to confirm the nature of this set, as a collection of genes that are required for essential functions such as cell division, metabolism, and development. Performing GO-term analysis also revealed that a number of the genes in this collection lacked sufficient annotation to be interpreted this way. We found four genes about which there is little to nothing known (*D2096.12*, *F56D5.2*, *T22B11.1*, and *Y54G2A.73*). For example, *F56D5.2* is a gene with no associated GO terms, no known protein domains, and no orthologs in other model organisms. These wholly uncharacterized genes are intriguing candidates which may help uncover new biological processes and biochemical pathways that are evidently fundamental to life for this organism.

### Examining expression patterns leads to discovery of genes involved in male fertility

The life stage-specific expression patterns (Supplementary File S4) provide some insight into the roles the genes in this collection play in development. Fifteen of the nineteen GOI are highly expressed in the early embryo and hermaphrodite gonad, which suggests that the gene product is passed on to the embryo from the parent. Five of these maternal genes also have elevated expression during late embryonic and larval stages, which suggests they are pleiotropic. The zygotic functions of these genes must be nonessential or else a zygotic lethal, rather than maternal-effect lethal, phenotype would be observed.

We also identified four genes that are most highly expressed in males and L4 hermaphrodites, as well as three genes that have prominent male expression in addition to characteristic maternal expression patterns. Mating assays confirmed that these male-expressed genes have an essential role in male fertility. Studies have shown that genes expressed in sperm are largely insensitive to RNAi (Fraser *et al.* 2000; Gönczy *et al.* 2000; Reinke *et al.* 2004; del Castillo-Olivares *et al.* 2009; Zhu *et al.* 2009; Ma *et al.* 2014), making these types of genes particularly difficult to identify in high-throughput RNAi screens. With the availability of RNA-seq data across different life stages for nearly every gene in the *C. elegans* genome (Hillier *et al.* 2009; Gerstein *et al.* 2010, 2014; Boeck *et al.* 2016; Tintori *et al.* 2016; Packer *et al.* 2019), screening for characteristic gene expression patterns may be a useful approach for identifying sterile and maternal-effect lethal genes that remain to be discovered.

We propose that the seven male-expressed genes are involved in sperm production and/or function (Table 5). These genes are mostly uncharacterized, and this is the first reporting of their involvement in male fertility. While the mutant hermaphrodites lay unfertilized oocytes (5 genes) or dead eggs (2 genes), this phenotype could be rescued in 14 of the 16 alleles by the introduction of wild-type sperm through mating. The two alleles that could not be rescued had allele pairs in the same complementation groups that were rescued in the mating assay. One of these discrepancies, between *F56D5.2*(*t1744*) and *F56D5.2*(*t1791*), was resolved when we found a second mutation in a nearby essential gene that was likely responsible for the inability of one strain to be rescued (data not shown). The presence of additional lethal mutations in the genome is unsurprising given the nature of chemical mutagenesis, and it reinforces the advantage of having multiple alleles for a gene when interpreting mutant phenotypes.

### Interpreting terminal phenotypes of maternal-effect lethal mutants

The catalog of terminal phenotypes (Supplementary File S5) created in this study provides a window into the roles the maternal-effect genes play in development. Some of these phenotypes corroborate previously observed phenotypes from RNAi studies. For example, RNAi knockdown experiments have shown that DLAT-1 is an enzyme involved in metabolic processes required for cell division in one-cell *C. elegans* embryos (Rahman *et al.* 2014). We uncovered two alleles of *dlat-1* in this study (*t2035* and *t2056*) in which most embryos arrest at the one- to four-cell stage (Figure 3B). The mutant alleles presented here can confirm previously reported phenotypes and serve as new genetic tools for continuing the study of essential gene function.

We also identified alleles for six genes that exhibit an OID phenotype, resulting in embryos that filled the eggshell completely or burst in distilled water. More than 100 genes have been identified in RNAi screens as important for the osmotic integrity of developing embryos (reviewed in Stein and Golden 2018). Some of these genes have roles in lipid metabolism (Rappleye *et al.* 2003; Benenati *et al.* 2009), cellular trafficking (Rappleye *et al.* 1999), and chitin synthesis (Johnston *et al.* 2006). Four of the six genes identified with OID mutants in this study have been previously implicated in osmotic sensitivity: *dgtr-1* is involved in lipid biosynthesis (Carvalho *et al.* 2011; Olson *et al.* 2012), *trcs-1* is involved in lipid metabolism and membrane trafficking (Green *et al.* 2011); *perm-5* is predicted to have lipid binding activity; and *F21D5.1* is an ortholog of human PGM3, an enzyme involved in the hexosamine pathway which generates substrates for chitin synthase. We found OID mutants for two additional genes that were not previously characterized with this phenotype, *bckd-1A* and *D2096.12*. *bckd-1A* is a component of the branched-chain alpha-keto dehydrogenase complex, which is involved in fatty acid biosynthesis (Kniazeva *et al.* 2004); this may be indicative of a role in generating or maintaining the lipid-rich permeability barrier. *D2096.12* is a *Caenorhabditis*-specific gene with no known protein domains. Elucidating the function of this uncharacterized gene may lead to new insights about the biochemistry of eggshell formation and permeability in *C. elegans* embryos.

Most of the mutant strains we examined with DIC microscopy arrested around the 100- to 200-cell stage as a seemingly disorganized group of cells (*e.g.*, Figure 3C). Others developed into two-fold or later stage embryos that moved inside the eggshell but did not hatch (*e.g.*, Figure 3D). The terminal phenotypes documented here reveal how long the embryo can persist without the maternal contribution of gene products, and the developmental defects that ensue. Future studies might make use of fluorescent markers and automated cell lineage tracking (*e.g.*, Thomas *et al.* 1996; Schnabel *et al.* 1997; Bao *et al.* 2006; Wang *et al.* 2019) as well as single-cell transcriptome data (Tintori *et al.* 2016; Packer *et al.* 2019) to further investigate these essential genes.

### Relevance beyond *C. elegans*

In this collection of 58 putative essential genes, there are 47 genes (81%) with human orthologs; a two-fold enrichment when compared to all *C. elegans* genes, 41% of which have human orthologs (Kim *et al.* 2018). This is in line with previous findings that essential genes are more often phylogenetically conserved than nonessential genes (Hughes 2002; Jordan *et al.* 2002; Georgi *et al.* 2013). Essential genes in model organisms are often associated with human diseases (Culetto and Sattelle 2000; Silverman *et al.* 2009; Dickerson *et al.* 2011; Qin *et al.* 2018), making the alleles identified in this study potentially relevant to understanding human health. Indeed, there are OMIM disease phenotypes associated with a number of the human orthologs listed in Table 2. Novel mutant alleles in *C. elegans* may help us better understand genetic disorders by providing new opportunities to interrogate gene function, explore genetic interactions, and screen prospective therapeutics.

Nematode-specific genes that are essential are important to nematode biology in general and are particularly relevant in parasitic nematology. We found three genes in our GOI list (*F56D5.2*, *perm-5*, and *T22B11.1*) that have orthologs in parasitic nematode species and not in other phyla. With growing anthelminthic drug resistance around the world (Jabbar *et al.* 2006), novel management strategies are needed to combat parasitic nematodes, which infect crops, livestock, and people worldwide (Nicol *et al.* 2011; Wolstenholme *et al.* 2004; Hotez *et al.* 2008). Essential genes are desirable targets for drug development, yet identifying such genes in parasites experimentally is difficult (Kumar *et al.* 2007; Doyle *et al.* 2010). Thus, as a free-living nematode, *C. elegans* is a widely used model for genetically intractable parasitic species (Bürglin *et al.* 1998; Hashmi *et al.* 2001). Our identification of novel essential genes with orthologs in parasitic nematodes may provide new opportunities to explore management strategies.

It is our hope that the alleles and phenotypes presented here will serve as a starting point and guide future research to elucidate the specific roles these genes play in embryogenesis. All of the alleles presented in this study are available to the research community through the Caenorhabditis Genetics Center (cgc.umn.edu) and we anticipate they will serve as a valuable resource in the years to come. The wealth of material uncovered in this specific legacy collection will hopefully inspire similar explorations of other frozen mutant collections.

## Data availability

The raw sequence data from this study have been deposited in the NCBI Sequence Read Archive (SRA; ncbi.nlm.nih.gov/sra) under accession number PRJNA628853. Supplemental material is available at figshare: Supplementary File S1 (Detailed experimental methods from the generation of Collection B); Supplementary File S2 (Gene candidate selection, Alleles and associated publications, Common gene hits with deficiency mapping for each complementation group); Supplementary File S3 (CRISPR-Cas9 deletion alleles and associated sequences, GO terms and associated genes); Supplementary File S4 (Life stage-specific expression patterns); Supplementary File S5 (Terminal phenotypes of maternal-effect lethal embryos). Figshare DOI: https://doi.org/10.25386/genetics.14702139

## References

[jkab328-B1] Altschul SF , Gish W, Miller W, Myers EW, Lipman DJ. 1990. Basic local alignment search tool. J Mol Biol. 215:403–410. 2231712 10.1016/S0022-2836(05)80360-2

[jkab328-B2] Au V , Li-Leger E, Raymant G, Flibotte S, Chen G, et al 2019. CRISPR/Cas9 methodology for the generation of knockout deletions in *Caenorhabditis elegans*. G3 (Bethesda). 9:135–144. 30420468 10.1534/g3.118.200778 PMC6325907

[jkab328-B3] Ausländer S , Ausländer D, Fussenegger M. 2017. Synthetic biology—the synthesis of biology. Angew Chem Int Ed Engl. 56:6396–6419. 27943572 10.1002/anie.201609229

[jkab328-B4] Bao Z , Murray JI, Boyle T, Ooi SL, Sandel MJ, et al 2006. Automated cell lineage tracing in *Caenorhabditis elegans*. Proc Natl Acad Sci USA. 103:2707–2712. 16477039 10.1073/pnas.0511111103 PMC1413828

[jkab328-B5] Benenati G , Penkov S, Müller-Reichert T, Entchev EV, Kurzchalia TV. 2009. Two cytochrome P450s in *Caenorhabditis elegans* are essential for the organization of eggshell, correct execution of meiosis and the polarization of embryo. Mech Dev. 126:382–393. 19368796 10.1016/j.mod.2009.02.001

[jkab328-B6] Bischoff M , Schnabel R. 2006. A posterior centre establishes and maintains polarity of the *Caenorhabditis elegans* embryo by a Wnt-dependent relay mechanism. PLoS Biol. 4:e396. 17121454 10.1371/journal.pbio.0040396 PMC1637133

[jkab328-B7] Blumenstiel JP , Noll AC, Griffiths JA, Perera AG, Walton KN, et al 2009. Identification of EMS-induced mutations in *Drosophila melanogaster* by whole-genome sequencing. Genetics. 182:25–32. 19307605 10.1534/genetics.109.101998 PMC2674820

[jkab328-B8] Boeck ME , Huynh C, Gevirtzman L, Thompson OA, Wang G, et al 2016. The time-resolved transcriptome of *C. elegans*. Genome Res. 26:1441–1450. 27531719 10.1101/gr.202663.115 PMC5052054

[jkab328-B9] Bradley A , Anastassiadis K, Ayadi A, Battey JF, Bell C, et al 2012. The mammalian gene function resource: the international knockout mouse consortium. Mamm Genome. 23:580–586. 22968824 10.1007/s00335-012-9422-2 PMC3463800

[jkab328-B10] Brauchle M , Baumer K, Gönczy P. 2003. Differential activation of the DNA replication checkpoint contributes to asynchrony of cell division in *C. elegans* embryos. Curr Biol. 13:819–827. 12747829 10.1016/s0960-9822(03)00295-1

[jkab328-B11] Brenner S. 1974. The genetics of *Caenorhabditis elegans*. Genetics. 77:71–94. 4366476 10.1093/genetics/77.1.71 PMC1213120

[jkab328-B12] Bürglin TR , Lobos E, Blaxter ML. 1998. *Caenorhabditis elegans* as a model for parasitic nematodes. Int J Parasitol. 28:395–411. 9559358 10.1016/s0020-7519(97)00208-7

[jkab328-B13] Carvalho A , Olson SK, Gutierrez E, Zhang K, Noble LB, et al 2011. Acute drug treatment in the early *C. elegans* embryo. PLoS One. 6:e24656. 21935434 10.1371/journal.pone.0024656 PMC3173474

[jkab328-B14] C. Elegans Deletion Mutant Consortium. 2012. Large-scale screening for targeted knockouts in the *Caenorhabditis elegans* genome. G3 (Bethesda). 2:1415–1425. 23173093 10.1534/g3.112.003830 PMC3484672

[jkab328-B15] Clark DV , Rogalski TM, Donati LM, Baillie DL. 1988. The unc-22 (IV) region of *Caenorhabditis elegans*: genetic analysis of lethal mutations. Genetics. 119:345–353. 3396868 10.1093/genetics/119.2.345 PMC1203417

[jkab328-B16] Cockell MM , Baumer K, Gönczy P. 2004. Lis-1 is required for dynein-dependent cell division processes in *C. elegans* embryos. J Cell Sci. 117:4571–4582. 15331665 10.1242/jcs.01344

[jkab328-B17] Culetto E , Sattelle DB. 2000. A role for *Caenorhabditis elegans* in understanding the function and interactions of human disease genes. Hum Mol Genet. 9:869–877. 10767309 10.1093/hmg/9.6.869

[jkab328-B18] del Castillo-Olivares A , Kulkarni M, Smith HE. 2009. Regulation of sperm gene expression by the GATA factor ELT-1. Dev Biol. 333:397–408. 19591818 10.1016/j.ydbio.2009.06.044 PMC6334776

[jkab328-B19] Delattre M , Leidel S, Wani K, Baumer K, Bamat J, et al 2004. Centriolar SAS-5 is required for centrosome duplication in C. elegans. Nat Cell Biol. 6:656–664. 15232593 10.1038/ncb1146

[jkab328-B20] Denver DR , Morris K, Lynch M, Thomas WK. 2004. High mutation rate and predominance of insertions in the *Caenorhabditis elegans* nuclear genome. Nature. 430:679–682. 15295601 10.1038/nature02697

[jkab328-B21] Dickerson JE , Zhu A, Robertson DL, Hentges KE. 2011. Defining the role of essential genes in human disease. PLoS One. 6:e27368. 22096564 10.1371/journal.pone.0027368 PMC3214036

[jkab328-B22] Doitsidou M , Poole RJ, Sarin S, Bigelow H, Hobert O. 2010. *C. elegans* mutant identification with a one-step whole-genome-sequencing and SNP mapping strategy. PLoS One. 5:e15435. 21079745 10.1371/journal.pone.0015435 PMC2975709

[jkab328-B23] Doyle MA , Gasser RB, Woodcroft BJ, Hall RS, Ralph SA. 2010. Drug target prediction and prioritization: using orthology to predict essentiality in parasite genomes. BMC Genomics. 11:222. 20361874 10.1186/1471-2164-11-222 PMC2867826

[jkab328-B24] Feichtinger RE. 1995. Quantitative Analysis of Maternal Gene Functions of *Caenorhabditis elegans* [Ph.D. thesis]. Austria: University of Vienna.

[jkab328-B25] Fire A , Xu S, Montgomery MK, Kostas SA, Driver SE, et al 1998. Potent and specific genetic interference by double-stranded RNA in *Caenorhabditis elegans*. Nature. 391:806–811. 9486653 10.1038/35888

[jkab328-B26] Flibotte S , Edgley ML, Chaudhry I, Taylor J, Neil SE, et al 2010. Whole-genome profiling of mutagenesis in *Caenorhabditis elegans*. Genetics. 185:431–441. 20439774 10.1534/genetics.110.116616 PMC2881127

[jkab328-B27] Fraser AG , Kamath RS, Zipperlen P, Martinez-Campos M, Sohrmann M, et al 2000. Functional genomic analysis of *C. elegans* chromosome I by systematic RNA interference. Nature. 408:325–330. 11099033 10.1038/35042517

[jkab328-B28] Georgi B , Voight BF, Bućan M. 2013. From mouse to human: evolutionary genomics analysis of human orthologs of essential genes. PLoS Genet. 9:e1003484. 23675308 10.1371/journal.pgen.1003484 PMC3649967

[jkab328-B29] Gerstein MB , Rozowsky J, Yan K, Wang D, Cheng C, et al 2014. Comparative analysis of the transcriptome across distant species. Nature. 512:445–448. 25164755 10.1038/nature13424 PMC4155737

[jkab328-B30] Gerstein MB , Lu ZJ, Van Nostrand EL, Cheng C, Arshinoff BI, et al; modENCODE Consortium. 2010. Integrative analysis of the *Caenorhabditis elegans* genome by the modENCODE project. Science. 330:1775–1787. 21177976 10.1126/science.1196914 PMC3142569

[jkab328-B31] Giaever G , Chu AM, Ni L, Connelly C, Riles L, et al 2002. Functional profiling of the *Saccharomyces cerevisiae* genome. Nature. 418:387–391. 12140549 10.1038/nature00935

[jkab328-B32] Golden JW , Riddle DL. 1984. A pheromone-induced developmental switch in *Caenorhabditis elegans*: temperature-sensitive mutants reveal a wild-type temperature-dependent process. Proc Natl Acad Sci USA. 81:819–823. 6583682 10.1073/pnas.81.3.819 PMC344929

[jkab328-B33] Gönczy P , Schnabel H, Kaletta T, Amores AD, Hyman T, et al 1999. Dissection of cell division processes in the one cell stage *Caenorhabditis elegans* embryo by mutational analysis. J Cell Biol. 144:927–946. 10085292 10.1083/jcb.144.5.927 PMC2148205

[jkab328-B34] Gönczy P , Bellanger J, Kirkham M, Pozniakowski A, Baumer K, et al 2001. Zyg-8, a gene required for spindle positioning in *C. elegans*, encodes a doublecortin-related kinase that promotes microtubule assembly. Dev Cell. 1:363–375. 11702948 10.1016/s1534-5807(01)00046-6

[jkab328-B35] Gönczy P , Echeverri C, Oegema K, Coulson A, Jones SJ, et al 2000. Functional genomic analysis of cell division in *C. elegans* using RNAi of genes on chromosome III. Nature. 408:331–336. 11099034 10.1038/35042526

[jkab328-B36] Goodwin S , McPherson JD, McCombie WR. 2016. Coming of age: ten years of next-generation sequencing technologies. Nat Rev Genet. 17:333–351. 27184599 10.1038/nrg.2016.49 PMC10373632

[jkab328-B37] Green RA , Kao H, Audhya A, Arur S, Mayers JR, et al 2011. A high-resolution *C. elegans* essential gene network based on phenotypic profiling of a complex tissue. Cell. 145:470–482. 21529718 10.1016/j.cell.2011.03.037 PMC3086541

[jkab328-B38] Hashmi S , Tawe W, Lustigman S. 2001. *Caenorhabditis elegans* and the study of gene function in parasites. Trends Parasitol. 17:387–393. 11685900 10.1016/s1471-4922(01)01986-9

[jkab328-B39] Herman RK. 1978. Crossover suppressors and balanced recessive lethals in *Caenorhabditis elegans*. Genetics. 88:49–65. 631558 10.1093/genetics/88.1.49 PMC1213790

[jkab328-B40] Hillier LW , Marth GT, Quinlan AR, Dooling D, Fewell G, et al 2008. Whole-genome sequencing and variant discovery in *C. elegans*. Nat Methods. 5:183–188. 18204455 10.1038/nmeth.1179

[jkab328-B41] Hillier LW , Reinke V, Green P, Hirst M, Marra MA, et al 2009. Massively parallel sequencing of the polyadenylated transcriptome of *C. elegans*. Genome Res. 19:657–666. 19181841 10.1101/gr.088112.108 PMC2665784

[jkab328-B42] Hotez PJ , Brindley PJ, Bethony JM, King CH, Pearce EJ, et al 2008. Helminth infections: the great neglected tropical diseases. J Clin Invest. 118:1311–1321. 18382743 10.1172/JCI34261 PMC2276811

[jkab328-B43] Howell AM , Gilmour SG, Mancebo RA, Rose AM. 1987. Genetic analysis of a large autosomal region in *Caenorhabditis elegans* by the use of a free duplication. Genet Res. 49:207–213.

[jkab328-B44] Howell AM , Rose AM. 1990. Essential genes in the hDf6 region of chromosome I in *Caenorhabditis elegans*. Genetics. 126:583–592. 2249758 10.1093/genetics/126.3.583 PMC1204214

[jkab328-B45] Hughes TR. 2002. Yeast and drug discovery. Funct Integr Genomics. 2:199–211. 12192593 10.1007/s10142-002-0059-1

[jkab328-B46] Hutter H , Suh J. 2016. GExplore 1.4: an expanded web interface for queries on *Caenorhabditis elegans* protein and gene function. Worm. 5:e1234659. 28090394 10.1080/21624054.2016.1234659 PMC5190144

[jkab328-B47] Hutter H , Ng M, Chen N. 2009. GExplore: a web server for integrated queries of protein domains, gene expression and mutant phenotypes. BMC Genomics. 10:529. 19917126 10.1186/1471-2164-10-529 PMC2779824

[jkab328-B48] Jabbar A , Iqbal Z, Kerboeuf D, Muhammad G, Khan MN, et al 2006. Anthelmintic resistance: the state of play revisited. Life Sci. 79:2413–2431. 16979192 10.1016/j.lfs.2006.08.010

[jkab328-B49] Jaramillo-Lambert A , Fuchsman AS, Fabritius AS, Smith HE, Golden A. 2015. Rapid and efficient identification of *Caenorhabditis elegans* legacy mutations using Hawaiian SNP-based mapping and whole-genome sequencing. G3 (Bethesda). 5:1007–1019. 25740937 10.1534/g3.115.017038 PMC4426357

[jkab328-B50] Johnsen RC , Baillie DL. 1988. Formaldehyde mutagenesis of the eT1 balanced region in *Caenorhabditis elegans*: dose—response curve and the analysis of mutational events. Mutat Res. 201:137–147. 3419443 10.1016/0027-5107(88)90120-0

[jkab328-B51] Johnsen RC , Baillie DL. 1991. Genetic analysis of a major segment [LGV (left)] of the genome of *Caenorhabditis elegans*. Genetics. 129:735–752. 1752418 10.1093/genetics/129.3.735 PMC1204741

[jkab328-B52] Johnsen RC , Baillie DL, 1997. Mutation. In:Riddle DL, Blumenthal T, Meyer BJ, Priess JR, editors. C. Elegans II. Cold Spring Harbor, NY: Cold Spring Harbor Laboratory Press. p. 79–95. 21413245

[jkab328-B53] Johnston WL , Krizus A, Dennis JW. 2006. The eggshell is required for meiotic fidelity, polar-body extrusion and polarization of the *C. elegans* embryo. BMC Biol. 4:35. 17042944 10.1186/1741-7007-4-35 PMC1635065

[jkab328-B54] Jordan IK , Rogozin IB, Wolf YI, Koonin EV. 2002. Essential genes are more evolutionarily conserved than are nonessential genes in bacteria. Genome Res. 12:962–968. 12045149 10.1101/gr.87702 PMC1383730

[jkab328-B55] Kadandale P , Chatterjee I, Singson A. 2009. Germline transformation of *Caenorhabditis elegans* by injection. Microinjection. 518:123–133. 10.1007/978-1-59745-202-1_10 PMC2796118 19085141

[jkab328-B56] Kaitna S , Schnabel H, Schnabel R, Hyman AA, Glotzer M. 2002. A ubiquitin C-terminal hydrolase is required to maintain osmotic balance and execute actin-dependent processes in the early *C. elegans* embryo. J Cell Sci. 115:2293–2302. 12006614 10.1242/jcs.115.11.2293

[jkab328-B57] Kamath RS , Fraser AG, Dong Y, Poulin G, Durbin R, et al 2003. Systematic functional analysis of the *Caenorhabditis elegans* genome using RNAi. Nature. 421:231–237. 12529635 10.1038/nature01278

[jkab328-B58] Kemphues KJ , Kusch M, Wolf N. 1988. Maternal-effect lethal mutations on linkage group II of *Caenorhabditis elegans*. Genetics. 120:977–986. 3224814 10.1093/genetics/120.4.977 PMC1203589

[jkab328-B59] Kim W , Underwood RS, Greenwald I, Shaye DD. 2018. OrthoList 2: a new comparative genomic analysis of human and *Caenorhabditis elegans* genes. Genetics. 210:445–461. 30120140 10.1534/genetics.118.301307 PMC6216590

[jkab328-B60] Kniazeva M , Crawford QT, Seiber M, Wang C, Han M. 2004. Monomethyl branched-chain fatty acids play an essential role in *Caenorhabditis elegans* development. PLoS Biol. 2:e257. 15340492 10.1371/journal.pbio.0020257 PMC514883

[jkab328-B61] Kumar S , Chaudhary K, Foster JM, Novelli JF, Zhang Y, et al 2007. Mining predicted essential genes of *Brugia malayi* for nematode drug targets. PLoS One. 2:e1189. 18000556 10.1371/journal.pone.0001189 PMC2063515

[jkab328-B62] Langenhan T , Prömel S, Mestek L, Esmaeili B, Waller-Evans H, et al 2009. Latrophilin signaling links anterior-posterior tissue polarity and oriented cell divisions in the *C. elegans* embryo. Dev Cell. 17:494–504. 19853563 10.1016/j.devcel.2009.08.008 PMC2819344

[jkab328-B63] Li H , Durbin R. 2009. Fast and accurate short read alignment with Burrows–Wheeler transform. Bioinformatics. 25:1754–1760. 19451168 10.1093/bioinformatics/btp324 PMC2705234

[jkab328-B64] Li H , Handsaker B, Wysoker A, Fennell T, Ruan J, et al; 1000 Genome Project Data Processing Subgroup. 2009. The sequence alignment/map format and SAMtools. Bioinformatics. 25:2078–2079. 19505943 10.1093/bioinformatics/btp352 PMC2723002

[jkab328-B65] Li Z , Vizeacoumar FJ, Bahr S, Li J, Warringer J, et al 2011. Systematic exploration of essential yeast gene function with temperature-sensitive mutants. Nat Biotechnol. 29:361–367. 21441928 10.1038/nbt.1832 PMC3286520

[jkab328-B66] Ma X , Zhu Y, Li C, Xue P, Zhao Y, et al 2014. Characterisation of *Caenorhabditis elegans* sperm transcriptome and proteome. BMC Genomics. 15:168. 24581041 10.1186/1471-2164-15-168 PMC4028957

[jkab328-B67] McKim KS , Heschl MF, Rosenbluth RE, Baillie DL. 1988. Genetic organization of the unc-60 region in *Caenorhabditis elegans*. Genetics. 118:49–59. 8608931 10.1093/genetics/118.1.49 PMC1203265

[jkab328-B68] McKim KS , Starr T, Rose AM. 1992. Genetic and molecular analysis of the dpy-14 region in *Caenorhabditis elegans*. Mol Gen Genet. 233:241–251. 1603066 10.1007/BF00587585

[jkab328-B69] Mello CC , Kramer JM, Stinchcomb D, Ambros V. 1991. Efficient gene transfer in *C. elegans*: extrachromosomal maintenance and integration of transforming sequences. EMBO J. 10:3959–3970. 1935914 10.1002/j.1460-2075.1991.tb04966.x PMC453137

[jkab328-B70] Meneely PM , Herman RK. 1979. Lethals, steriles and deficiencies in a region of the X chromosome of *Caenorhabditis elegans*. Genetics. 92:99–115. 574105 10.1093/genetics/92.1.99 PMC1213963

[jkab328-B71] Metzker ML. 2010. Sequencing technologies—the next generation. Nat Rev Genet. 11:31–46. 19997069 10.1038/nrg2626

[jkab328-B72] Molin L , Schnabel H, Kaletta T, Feichtinger R, Hope IA, et al 1999. Complexity of developmental control: analysis of embryonic cell lineage specification in *Caenorhabditis elegans* using pes-1 as an early marker. Genetics. 151:131–141. 9872954 10.1093/genetics/151.1.131 PMC1460461

[jkab328-B73] Nicol JM , Turner SJ, Coyne DL, Nijs LD, Hockland S, et al 2011. Current nematode threats to world agriculture. In: Jones J, Gheysen G., Fenoll C, editors. Genomics and Molecular Genetics of Plant-Nematode Interactions. Dordrecht: Springer. p. 21–43.

[jkab328-B74] Nieto C , Almendinger J, Gysi S, Gómez-Orte E, Kaech A, et al 2010. Ccz-1 mediates the digestion of apoptotic corpses in *C. elegans*. J Cell Sci. 123:2001–2007. 20519582 10.1242/jcs.062331

[jkab328-B75] Nordström KJ , Albani MC, James GV, Gutjahr C, Hartwig B, et al 2013. Mutation identification by direct comparison of whole-genome sequencing data from mutant and wild-type individuals using k-mers. Nat Biotechnol. 31:325–330. 23475072 10.1038/nbt.2515

[jkab328-B76] Norris AD , Kim H, Colaiacovo MP, Calarco JA. 2015. Efficient genome editing in *Caenorhabditis elegans* with a toolkit of dual-marker selection cassettes. Genetics. 201:449–458. 26232410 10.1534/genetics.115.180679 PMC4596661

[jkab328-B77] Olson SK , Greenan G, Desai A, Müller-Reichert T, Oegema K. 2012. Hierarchical assembly of the eggshell and permeability barrier in *C. elegans*. J Cell Biol. 198:731–748. 22908315 10.1083/jcb.201206008 PMC3514041

[jkab328-B78] Packer JS , Zhu Q, Huynh C, Sivaramakrishnan P, Preston E, et al 2019. A lineage-resolved molecular atlas of *C. elegans* embryogenesis at single-cell resolution. Science. 365:eaax1971. 31488706 10.1126/science.aax1971 PMC7428862

[jkab328-B79] Qin Z , Johnsen R, Yu S, Chu JS, Baillie DL, et al 2018. Genomic identification and functional characterization of essential genes in *Caenorhabditis elegans*. G3 (Bethesda). 8:981–997. 29339407 10.1534/g3.117.300338 PMC5844317

[jkab328-B80] Rahman MM , Rosu S, Joseph-Strauss D, Cohen-Fix O. 2014. Down-regulation of tricarboxylic acid (TCA) cycle genes blocks progression through the first mitotic division in *Caenorhabditis elegans* embryos. Proc Natl Acad Sci USA. 111:2602–2607. 24550289 10.1073/pnas.1311635111 PMC3932911

[jkab328-B81] Rappleye CA , Paredez AR, Smith CW, McDonald KL, Aroian RV. 1999. The coronin-like protein POD-1 is required for anterior–posterior axis formation and cellular architecture in the nematode *Caenorhabditis elegans*. Genes Dev. 13:2838–2851. 10557211 10.1101/gad.13.21.2838 PMC317117

[jkab328-B82] Rappleye CA , Tagawa A, Bot NL, Ahringer J, Aroian RV. 2003. Involvement of fatty acid pathways and cortical interaction of the pronuclear complex in *Caenorhabditis elegans* embryonic polarity. BMC Dev Biol. 3:8. 14527340 10.1186/1471-213X-3-8 PMC270048

[jkab328-B83] Reinke V , San Gil I, Ward S, Kazmer K. 2004. Genome-wide germline-enriched and sex-biased expression profiles in *Caenorhabditis elegans*. Development. 131:311–323. 14668411 10.1242/dev.00914

[jkab328-B84] Rogalski TM , Moerman DG, Baillie DL. 1982. Essential genes and deficiencies in the unc-22 IV region of *Caenorhabditis elegans*. Genetics. 102:725–736. 7187364 10.1093/genetics/102.4.725 PMC1201969

[jkab328-B85] Samson ML , Lisbin MJ, White K. 1995. Two distinct temperature-sensitive alleles at the Elav locus of Drosophila are suppressed nonsense mutations of the same tryptophan codon. Genetics. 141:1101–1111. 8582616 10.1093/genetics/141.3.1101 PMC1206833

[jkab328-B86] Sarin S , Prabhu S, O'meara MM, Pe'er I, Hobert O. 2008. *Caenorhabditis elegans* mutant allele identification by whole-genome sequencing. Nat Methods. 5:865–867. 18677319 10.1038/nmeth.1249 PMC2574580

[jkab328-B87] Schnabel R , Hutter H, Moerman D, Schnabel H. 1997. Assessing normal embryogenesis in *Caenorhabditis elegans* using a 4D microscope: variability of development and regional specification. Dev Biol. 184:234–265. 9133433 10.1006/dbio.1997.8509

[jkab328-B88] Schneeberger K , Ossowski S, Lanz C, Juul T, Petersen AH, et al 2009. SHOREmap: simultaneous mapping and mutation identification by deep sequencing. Nat Methods. 6:550–551. 19644454 10.1038/nmeth0809-550

[jkab328-B89] Schneeberger K , Weigel D. 2011. Fast-forward genetics enabled by new sequencing technologies. Trends Plant Sci. 16:282–288. 21439889 10.1016/j.tplants.2011.02.006

[jkab328-B90] Shi J , Wang E, Milazzo JP, Wang Z, Kinney JB, et al 2015. Discovery of cancer drug targets by CRISPR-Cas9 screening of protein domains. Nat Biotechnol. 33:661–667. 25961408 10.1038/nbt.3235 PMC4529991

[jkab328-B91] Silverman GA , Luke CJ, Bhatia SR, Long OS, Vetica AC, et al 2009. Modeling molecular and cellular aspects of human disease using the nematode *Caenorhabditis elegans*. Pediatr Res. 65:10–18. 18852689 10.1203/PDR.0b013e31819009b0 PMC2731241

[jkab328-B92] Smith DR , Quinlan AR, Peckham HE, Makowsky K, Tao W, et al 2008. Rapid whole-genome mutational profiling using next-generation sequencing technologies. Genome Res. 18:1638–1642. 18775913 10.1101/gr.077776.108 PMC2556265

[jkab328-B93] Smith HE , Fabritius AS, Jaramillo-Lambert A, Golden A. 2016. Mapping challenging mutations by whole-genome sequencing. G3 (Bethesda). 6:1297–1304. 26945029 10.1534/g3.116.028316 PMC4856081

[jkab328-B94] Sonneville R , Gönczy P. 2004. Zyg-11 and cul-2 regulate progression through meiosis II and polarity establishment in *C. elegans*. Development. 131:3527–3543. 15215208 10.1242/dev.01244

[jkab328-B95] Sönnichsen B , Koski LB, Walsh A, Marschall P, Neumann B, et al 2005. Full-genome RNAi profiling of early embryogenesis in *Caenorhabditis elegans*. Nature. 434:462–469. 15791247 10.1038/nature03353

[jkab328-B96] Srivatsan A , Han Y, Peng J, Tehranchi AK, Gibbs R, et al 2008. High-precision, whole-genome sequencing of laboratory strains facilitates genetic studies. PLoS Genet. 4:e1000139. 18670626 10.1371/journal.pgen.1000139 PMC2474695

[jkab328-B97] Stein KK , Golden A. 2018. The *C. elegans* eggshell. WormBook. 2018:1–36. 10.1895/wormbook.1.179.1 PMC5603424 26715360

[jkab328-B98] Stewart HI , O'Neil NJ, Janke DL, Franz NW, Chamberlin HM, et al 1998. Lethal mutations defining 112 complementation groups in a 4.5 mb sequenced region of *Caenorhabditis elegans* chromosome III. Mol Gen Genet. 260:280–288. 9862482 10.1007/pl00013816

[jkab328-B99] ΘThe C. elegans Sequencing Consortium, 1998. Genome sequence of the nematode *C. elegans*: a platform for investigating biology. Science. 282:2012–2018. 9851916 10.1126/science.282.5396.2012

[jkab328-B100] Thomas C , DeVries P, Hardin J, White J. 1996. Four-dimensional imaging: computer visualization of 3D movements in living specimens. Science. 273:603–607. 8662545 10.1126/science.273.5275.603

[jkab328-B101] Thomas PD , Campbell MJ, Kejariwal A, Mi H, Karlak B, et al 2003. PANTHER: a library of protein families and subfamilies indexed by function. Genome Res. 13:2129–2141. 12952881 10.1101/gr.772403 PMC403709

[jkab328-B102] Thompson O , Edgley M, Strasbourger P, Flibotte S, Ewing B, et al 2013. The million mutation project: a new approach to genetics in *Caenorhabditis elegans*. Genome Res. 23:1749–1762. 23800452 10.1101/gr.157651.113 PMC3787271

[jkab328-B103] Tintori SC , Nishimura EO, Golden P, Lieb JD, Goldstein B. 2016. A transcriptional lineage of the early *C. elegans* embryo. Dev Cell. 38:430–444. 27554860 10.1016/j.devcel.2016.07.025 PMC4999266

[jkab328-B104] Varshney GK , Lu J, Gildea DE, Huang H, Pei W, et al 2013. A large-scale zebrafish gene knockout resource for the genome-wide study of gene function. Genome Res. 23:727–735. 23382537 10.1101/gr.151464.112 PMC3613589

[jkab328-B105] Vatcher GP , Thacker CM, Kaletta T, Schnabel H, Schnabel R, et al 1998. Serine hydroxymethyltransferase is maternally essential in *Caenorhabditis elegans*. J Biol Chem. 273:6066–6073. 9497323 10.1074/jbc.273.11.6066

[jkab328-B106] von Tobel L , Mikeladze-Dvali T, Delattre M, Balestra FR, Blanchoud S, et al 2014. SAS-1 is a C2 domain protein critical for centriole integrity in *C. elegans*. PLoS Genet. 10:e1004777. 25412110 10.1371/journal.pgen.1004777 PMC4238951

[jkab328-B107] Vyas VK , Barrasa MI, Fink GR. 2015. A *Candida albicans* CRISPR system permits genetic engineering of essential genes and gene families. Sci Adv. 1:e1500248. 25977940 10.1126/sciadv.1500248 PMC4428347

[jkab328-B108] Wang S , Ochoa SD, Khaliullin RN, Gerson-Gurwitz A, Hendel JM, et al 2019. A high-content imaging approach to profile *C. elegans* embryonic development. Development. 146:dev174029. 30890570 10.1242/dev.174029 PMC6467471

[jkab328-B109] Winzeler EA , Shoemaker DD, Astromoff A, Liang H, Anderson K, et al 1999. Functional characterization of the *S. cerevisiae* genome by gene deletion and parallel analysis. Science. 285:901–906. 10436161 10.1126/science.285.5429.901

[jkab328-B110] Wolstenholme AJ , Fairweather I, Prichard R, von Samson-Himmelstjerna G, Sangster NC. 2004. Drug resistance in veterinary helminths. Trends Parasitol. 20:469–476. 15363440 10.1016/j.pt.2004.07.010

[jkab328-B111] Yu L , Castillo LP, Mnaimneh S, Hughes TR, Brown GW. 2006. A survey of essential gene function in the yeast cell division cycle. Mol Biol Cell. 17:4736–4747. 16943325 10.1091/mbc.E06-04-0368 PMC1635385

[jkab328-B112] Zhang M , Wang C, Otto TD, Oberstaller J, Liao X, et al 2018. Uncovering the essential genes of the human malaria parasite *Plasmodium falciparum* by saturation mutagenesis. Science. 360:eaap7847. 29724925 10.1126/science.aap7847 PMC6360947

[jkab328-B113] Zhu G , Salazar G, Zlatic SA, Fiza B, Doucette MM, et al 2009. SPE-39 family proteins interact with the HOPS complex and function in lysosomal delivery. Mol Biol Cell. 20:1223–1240. 19109425 10.1091/mbc.E08-07-0728 PMC2642739

[jkab328-B114] Zuryn S , Gras SL, Jamet K, Jarriault S. 2010. A strategy for direct mapping and identification of mutations by whole-genome sequencing. Genetics. 186:427–430. 20610404 10.1534/genetics.110.119230 PMC2940307

